# Anaesthesia for pigs, sheep, goats and cattle involved in biomedical research: FELASA working group guidelines—Parts I–IV

**DOI:** 10.1177/00236772261442027

**Published:** 2026-06-11

**Authors:** Sabine J. Bischoff, Gabrielle C. Musk, Alenka Seliškar, Daniela Casoni, Stéphanie De Vleeschauwer, R. Eddie Clutton

**Affiliations:** 1 Heinrich-Heine-University, Medical Faculty, Institute of Laboratory Animal Science, Duesseldorf, Germany; 2 School of Veterinary Medicine, Murdoch University, Perth, Australia and School of Human Sciences, University of Western Australia, Perth, Australia; 3 University of Ljubljana, Veterinary Faculty, Ljubljana, Slovenia; 4 University of Bern, Faculty of Medicine, Experimental Animal Center, Experimental Animal Facility, Bern, Switzerland; 5 Laboratory Animal Center, KU Leuven, Leuven, Belgium; 6 Large Animal Research and Imaging Facility, Roslin Institute, University of Edinburgh, Roslin, Midlothian, UK

**Keywords:** FELASA, guidelines, recommendations, anaesthesia, analgesia, acclimatization, habituation, training, restraint, monitoring, pain, assessment, pigs, sheep, goats, cattle, laboratory, experimental, research

## Abstract

Recommendations for safe anaesthesia are common in medical practice, have been formulated for traditional laboratory species, i.e. rodents, but do not exist for laboratory pigs, sheep, goats and cattle. The guidelines presented here were commissioned by the Federation of European Laboratory Animal Science Associations (FELASA) and serve to underscore EU Directive 2010/63/EU (Article 14) which require that, ‘procedures are carried out with general or local anaesthesia and analgesia or another appropriate method to ensure pain, suffering and distress are kept to a minimum’. The guidelines are based on a review of: (i) medical and veterinary medical guidelines promoting safe anaesthetic practice; (ii) scientific literature pertaining to anaesthesia and analgesia in pigs, sheep, goats and cattle; and (iii) a consideration of the ethical, legal and scientific requirements when anaesthetizing laboratory animals. The guidelines aim to make recommendations for the provision of safe, practical and effective anaesthesia and analgesia to laboratory pigs and ruminants. Recommended standards for sedation and restraint (I), general principles of anaesthesia (II), monitoring anaesthesia (III) and pain assessment (IV) in the same species have been described by this working group (WG) and are set out in four parts in this document.

## Introduction

European legislation protecting animals in research^1^ is based on the ‘three-Rs’ principle^2^ which proposes that studies from which animals cannot be replaced (by *in vitro* or *in silico* methods) must use the fewest animals required (reduction) to achieve scientific goals, while optimizing their life experiences (refinement). Reduction may be achieved through sound scientific methodology (see NC3Rs Experimental Design Assistant), appropriate statistical analyses and observation of the PREPARE (Planning Research and Experimental Procedures on Animals: Recommendations for Excellence) guidelines.^3^

The EU Directive 2010/63/EU Article 3 (1) defines a ‘procedure’ as any use of an animal for experimental purposes which may cause the animal a level of pain, suffering, distress or lasting harm equivalent to, or greater than, that caused by the introduction of a needle in accordance with good veterinary practice.^1^ However, some national welfare acts or directives use slightly different definitions, e.g. the Animal (Scientific Procedures) Act 1986 of the UK also regards ‘administering an anaesthetic, an analgesic or other measure to sedate or dull the perception of pain’ as a procedure.^4^ To avoid confusion, the EU Directive 2010/63/EU definition will be used in the following recommendations and the term ‘procedure’ avoided when the provision of anaesthesia and/or analgesia is being described.

These guidelines were commissioned by the Federation of European Laboratory Animal Science Associations (FELASA) and comply with their terms of reference. The guidelines are based on a comprehensive review of the scientific literature pertaining to: (i) behavioural preparation and restraint; (ii) general principles of anaesthesia; (iii) anaesthesia monitoring; and (iv) pain assessment in laboratory pigs, sheep, cattle and goats. Their goal is to assist compliance with EU Directive 2010/63 (Article 14) which expects that ‘procedures are carried out with general or local anaesthesia and analgesia *or another appropriate method* to ensure pain, suffering and distress are kept to a minimum’. In more general terms, these guidelines aim to describe anaesthetic and analgesic approaches that optimize animal welfare and experimental data quality. Where the available evidence base is weak and incomplete, the recommendations have been underpinned by Working Group (WG) members’ experiences.

The challenge in making broadly applicable recommendations in the face of wide variation in, amongst other things, personnel competency and facility provision is not uncommonly faced by medical specialities attempting to establish world-wide practice standards. For this reason, the current document uses the standardized language of the World Health Organization (WHO) and the World Federation of Societies of Anaesthesiologists^5^ to denote three levels of standard: highly recommended; recommended; and suggested. Highly recommended standards are the minimum expected standards, i.e. the functional equivalent of mandatory standards. Recommended and suggested standards should be practiced when resources allow and if appropriate for the procedure, experiment and the animal(s) involved. In all circumstances, the aim is to attain the highest possible standards, preferably exceeding those outlined in these guidelines. In resource-poor settings where highly recommended standards cannot be met, the provision of anaesthesia for experimental procedures should be recognized as unsafe and, in undermining experimental refinement, unacceptable. In these circumstances, those responsible for facility provision and staff training must make every effort to ensure that highly recommended standards are met as rapidly as possible.

An understanding of animal behaviour is a prerequisite to: (a) the humane (stress-free) application of; and (b) understanding the scientific consequences of (i) behavioural preparation, i.e. acclimatisation, habituation, familiarization, socialization and training, (ii) physical restraint and (iii) aversive experimental requirements, e.g. isolation or separation. It also benefits pain assessment and management. While species-level understanding is the basic requirement, it must be noted that normal behaviour in any given animal will be affected by breed, sex, age and previous experiences. Farm animal behaviour and welfare science are highly specialized subjects and beyond the scope of this article. Named veterinary surgeons (NVSs) or designated veterinarians (DVs) should consult appropriate experts whenever they feel their expertise is inadequate.

Throughout these recommendations the four species, pigs, sheep, goats and cattle, will be referred to specifically, or as ‘agricultural’ species, production animals, livestock or farm(ed) animals. However, the recommendations do not extend to farmed poultry or fish. The term ‘cattle’ refers to all *bovidae*; the term ‘calves’ is used when referring specifically to young (pre-ruminant) cattle. The term ‘ruminant’ is also used for cattle, sheep and goats with a functioning rumen. The terms ‘pigs’, ‘sheep’ and ‘goats’ also encompasses ‘piglets’, ‘lambs’ and ‘kids’, which are differentiated when necessary. Minipigs and other *suidae* are only differentiated from other pigs when relevant.

## Part I: Behavioural preparation and restraint for pigs, sheep, goats and cattle involved in biomedical research

This section provides recommendations for the: (i) behavioural preparation (several distinct steps may be required to optimize an animal’s behavioural state before enrolment in experiments; these are acclimatization, familiarization, habituation, socialization and training; in these guidelines, the term ‘behavioural preparation’ will be used to describe one or a combination of these steps); and (ii) safe, practical, effective and humane restraint of farm animals allowing experimental procedures to be accomplished, with or without anaesthetics and analgesics. The need for an understanding of animal behaviour as a prerequisite to behavioural preparation and restraint has already been stated.

### General (all species) considerations

#### Behavioural preparation

##### Introduction

Introducing farm animals to the laboratory environment raises several challenges^6^ but, importantly, produces adverse physiological and psychological effects which vary in severity and duration and may be collectively termed as ‘stress’, i.e. ‘the sum of biological reactions to adverse physical, mental, or emotional, internal or external stimuli, that tends to disturb the homeostasis of an organism’.^7^ These biological reactions are highly undesirable as they are likely to: (a) confound data; (b) complicate day-to-day animal handling^8^; and (c) may have adverse health consequences such as decreased immunity and thus disease susceptibility.^9^ The stress of introducing farm animals to the laboratory arises from (a) transport and (b) the new (unfamiliar) environment.

##### Animal sourcing and transport

Farms or other facilities supplying ruminants and pigs to laboratories should meet criteria set by the destination laboratory and confirmed by periodic inspection by the facility’s NVS or DV. Recommended criteria are: demonstration of high health status^6^ and high welfare status; high levels of animal–stockperson interaction; common environmental and husbandry features (with laboratory); brief transit time to facility. Such criteria are necessary for making accurate accumulative severity assessments^10^ for animals obtained outwith the facility. These, in turn, are required for informed decision-making with respect to the animals’: (i) allocation to studies of different severities; (ii) re-use; or (iii) scheduled killing. For these reasons, obtaining inexpensive cull animals from public markets is strongly discouraged.

The effects of transport on pigs, sheep and calves are well documented.^11^ In general, transport raises cortisol and body temperature and invokes cardiovascular and behavioural changes.^12,13^ Identified transport stressors are duration of transport, separation from familiar conditions, container and vehicle design, food and water supply and driver attitude.^11^ Therefore, transport stress can be minimized by limiting transport time, minimizing animal numbers per transport, considering species-related environmental conditions and ensuring that all staff are properly trained.^11^ Transport stress can be avoided by using facility-bred animals with laboratory adjacency. Numerous features of the laboratory environment will be entirely unfamiliar to most animals sourced outwith research facilities (except, perhaps, for purpose-bred minipigs). Animals will have been reared under conditions ranging from intensive, densely stocked indoor systems (pigs) to extensive outdoor ranges where animals (sheep) will have had little human contact. This contrasts with the regulated, usually indoor, laboratory conditions in which most environmental features are legally prescribed, closely managed, but nevertheless unfamiliar: the new environment, diet, bedding, lighting regimes, temperature and humidity, drinking facilities, pen- or room-mates and the (human) care and scientific staff with which they will interact.

##### Acclimatization

Acclimatization allows out-sourced farm animals to adapt to their new environment, achieving physiological, psychological and nutritional stabilization. Its specific purpose is to allow time for the neuro-endocrinological changes arising from transport stress and neophobia to resolve, so that animals may be enrolled on study as soon as possible. During acclimatization some baseline physiological and behavioural variables such as food and water intake can be determined; relevant biological samples can be collected; and animal health confirmed before study. Importantly, successful acclimatisation is a prerequisite to subsequent familiarization, habituation and training, if necessary. Successful habituation and training, in turn, may result in animals allowing some procedures to be conducted with minimal physical or pharmacological restraint. (In these recommendations, the term ‘pharmacological restraint’ is used to mean any drug or drug combination, e.g. tranquilizers, anxiolytics, sedative-hypnotics, anaesthetics and analgesics, that are used to reduce, ideally preclude, the need for (and stress of) physical restraint and allow scientific procedures to be carried out.). When sedation or general anaesthesia are unavoidable, minimal stress levels are still necessary because anxiety is a lethal risk factor in medical anaesthesia.^14^ Consequently, acclimatization, habituation and training can be seen as major contributors to experimental refinement, which is in accordance with European legislation protecting animals in research.^1^ Finally, the ARRIVE 2.0 guidelines (Animal Research: Reporting of In Vivo Experiments) ask for a description of ‘Welfare-related assessments and interventions that were carried out prior to, during, or after the experiment’ in the Essential 10 list, Section 9c. This supports the notion that reporting details of acclimatisation, habituation and training contributes to experimental reproducibility.^15^

Acclimatization methods significantly affect study outcomes^7^ so must be carefully considered. Its fundamental advantages, i.e. stress reduction, optimized animal welfare and data quality, outweigh the main disadvantages (increased costs from delayed study onset and increased time commitment from scientists and animal facility staff) and support the recommendation that *all* animals undergo acclimatization.^7,16^ However, methods and duration will depend on numerous factors, with ‘source’, along with species, age, sex, breed and function, probably being the most important. It seems intuitive that laboratory-bred minipigs will require less acclimatization than freshly weaned growers from a commercial herd, while a laboratory-bred sheep will not require the intense, possibly futile attempts to acclimatize an aged ewe which has never been housed. Other factors determining the elements of acclimatization include the nature and duration of the experimental work (experimental outputs) and recovery versus non-recovery procedures. The feasibility of acclimatisation depends on the laboratory infrastructure and the experience of the personnel involved. People competent in acclimatizing and/or restraining animals are important in minimizing the stress associated with human interaction^17^ and so represent experimental refinement. Therefore, they should be recruited as a matter of priority. Acclimatization also allows the collection and analysis of baseline blood, urine and other samples to facilitate the later interpretation of treatment effects. Acclimatisation should last until its pre-defined objectives are met and there is confidence that the study begins with each animal being biologically comparable and all animals being at similarly low levels of stress.

Ideally, animals would be acclimatized, habituated and trained as promptly as possible, to undergo all necessary procedures without physical restraint or the need for drugs affecting scientific outcomes. This idyll is unrealistic and so study planning must establish beforehand the extent to which physical and/or pharmacological restraint will be necessary (practically) and permissible (ethically, legally and scientifically). While the primary goal of acclimatization is always to reduce stress to levels which will not obfuscate scientific outcomes, the methods used and periods they are applied for will depend on the predicted cost: benefit ratio of using options involving physical and/or pharmacological restraint. In the final analysis, habituation and especially training are time-consuming and expensive, while results may be variable and not always sustainable; adverse (noxious) experiences can rapidly reverse an animal’s procedural cooperativity.^18,19^

##### Familiarization and socialization

Familiarization and socialization are important elements of acclimatisation. Familiarization involves exposing animals to all personnel which they will interact with while on study. This includes those involved in cleaning and feeding, as well as those conducting procedures. The goal is to reduce fear, stress and anxiety and make animals comfortable with the proximity and actions of humans without necessarily altering their social behaviours. The advantages of familiarization are fear reduction, improved handling and improved welfare. Familiarization is achieved by exposing animals to frequent positive interactions, i.e. repeated gentle contact, gentle stroking, talking softly and offering food rewards.

Socialization, an extension of familiarization, actively encourages animals to engage socially with relevant laboratory personnel to produce affiliative behaviours and interactions. Socialization seeks to promote positive social behaviours in animals and develop relationships and interactions that go beyond mere tolerance. Human–animal socialization is particularly desirable in studies which may have aversive physiological, e.g. pain, or psychological outcomes. Subtle post-procedural behavioural changes are likely to be more apparent in socialized animals, which will facilitate pain (and severity) assessment.

##### Habituation

Habituation describes an animal’s behavioural adaptation to repeated or constant stimuli, resulting in a diminished response over time. This form of non-associative learning enables animals to filter irrelevant information and allocate cognitive resources more efficiently. Unlike acclimatization, which is primarily physiological, habituation is a behavioural process that allows animals to ignore non-threatening stimuli or those irrelevant to survival. Habituating animals to non-aversive activities such as thoracic auscultation, rectal temperature monitoring or ultrasonic examination, while time-consuming, will yield more representative data than the same examinations conducted using physical and/or pharmacological restraint. Habituation is necessary when minimizing the effects of human presence is critical, e.g. for unbiased observations of natural behaviours. It should be noted that animals will not habituate to extreme treatments. ‘Flooding’, i.e. where an individual is acutely exposed to potentially aversive stimuli at full intensity, without any gradual introduction, can be highly distressing and has no place in laboratory animal habituation.

##### Training

Training is an intentional, structured process in which animals associatively learn to enact specific behaviours through repeated exposure to cues and rewards. Unlike habituation, which involves a generalized reduction in response, training focuses on eliciting specific, project-focused behaviours. Positive reinforcement, using, for example, high-quality food rewards, is commonly used in training to reinforce the learning of behaviours that are beneficial for both the animal and laboratory staff. While animal training is a specialist activity and beyond the scope of this document^20^ information is available elsewhere for training laboratory pigs and sheep.^14,21^

##### Separation or isolation studies

Isolation, or single housing of animals after periods of group housing, causes agitation, anxiety and is extremely stressful in the social species, i.e. pigs,^22^ sheep,^23^ goats,^23^ calves^24^ and cattle^25^ so must be avoided unless there are compelling scientific, veterinary or practical justifications. Sexually mature boars are naturally solitary animals making single housing less important.

##### Non-recovery procedures

The need for acclimatization may be challenged when animals reared ‘off-site’ are required for non-recovery studies in which stress variables are unimportant. In these circumstances, anaesthetizing animals as soon as they reach the facility avoids problems with alternative stressors, e.g. prolonged solitary housing or mixing with ‘local’ animals. Mixing unfamiliar animals (of any species) upon arrival at research facilities should be avoided because the ensuing disruption of established social hierarchies causes stress.^26,27^ Whether such animals are delivered with a travelling ‘companion’ demands consideration. The benefits of pairing, which are likely to be greatest when long distances are involved, will be negated if the second animal is superfluous to study requirements, because it must either be killed or undergo the stress of being single. This option should only be considered when animals are obtained from trusted farms with high health status and reliable health records, which are located within brief transit times from the facility and when the experiment’s scientific outcomes are unaltered by transit stress. The risk of anaesthesia complications in animals with transport stress may be reduced by ensuring pre-anaesthetic medication contains anxiolytic as well as sedative components and delaying induction until their effects are apparent.

#### Physical restraint

Physical restraint methods that are commonly used under farm conditions may be inappropriate for laboratory animals as many will challenge refinement principles and/or affect study outcomes.^7^ In the UK and EU member states, codes of practice exist to provide guidance in interpreting and prosecuting legislation protecting the welfare of commercial pigs,^28,29^ sheep,^30^ goats^31^ and cattle.^32,33^ Details on handling and restraint contained within these codes must be regarded as the *minimum* requirement for restraining and handling these species under laboratory conditions.

An animal’s physical and biological reactions to various interventions, e.g. physical examination, research procedures, must be minimized for reasons of personnel and animal safety, animal welfare and data quality, respectively. This is achieved using one or a combination of the following methods: (i) behavioural preparation; (ii) physical restraint; and/or; (iii) drug administration (pharmacological restraint).

Physical restraint will be stressful for animals and personnel, particularly when the former are not accustomed to it and the latter are inexperienced in applying it. Repeated positive experiences with continual restraint attempts may result in habituation.^34,35^ Conversely, aversive attempts will make procedures increasingly difficult and may initiate ‘emotional contagion’. This occurs in animals which, upon perceiving conspecifics to be distressed, show signs of increased attentiveness, anxiety and fear themselves.^36^ The likelihood of ‘emotional contagion’ is enhanced by previous exposure to similar stressors.^36^ When emotional contagion is likely, social support, i.e. the presence of conspecifics, may prove beneficial, particularly if the animals possess a ‘low resistance coping style’.^37^ For these reasons, physical restraint methods alone must be used judiciously in the laboratory.

The selected restraint technique must ideally: (i) provide conditions that allow the intended procedure to be completed promptly without causing pain, suffering, distress or lasting harm to the animal; (ii) not interfere with the study objectives; (iii) be safely repeatable; (iv) be easily applied and; (v) not create excessive demands on laboratory staff in terms of time commitment and physical risk.^34,38,39^ If procedures allow, the ideal restraint method would involve trained animals voluntarily entering a familiar, non-threatening restraint device. It must be noted that the use of electrical devices that stun farmed animals for restraint and/or immobilization purposes is banned in the UK and Ireland.

#### Accessory techniques

Problems with behavioural preparation (time-consumption and expense), physical (stress) and pharmacological restraint (data effects) can be reduced by using data collection methods that limit the necessity for all three approaches.

##### Telemetry

Telemetry allows the continuous collection of physiological data from numerous animals simultaneously, e.g. control and treatment animals, which is unaffected by the confounding effects of human presence. Invasive (surgically implanted) and non-invasive (collars; jackets) technologies are available for pigs, sheep and calves. The pros and cons of telemetry in laboratory (but not farm) animals have been reviewed elsewhere.^40,41^

##### Thermometry

Microchip and infrared thermometry can be used to measure body temperature in pigs, provided baseline readings and correction factors are established. The former can also be used in sheep and goats. Both methods provide consistent measurements in individual animals and populations over time.^42^

##### Metabolism cages

Metabolism cages have been used for qualitative and quantitative studies requiring the simultaneous collection of urine and faeces while monitoring food and water intake. However, they severely limit mobility and invariably require animal isolation for extended periods (>12 hours). These conditions are highly stressful and while this may be ameliorated by acclimatization, they may nevertheless compromise data quality. The advantages of metabolism cages probably do not justify these disadvantages. Alternatives to metabolism cages that require individual housing but allow for olfactory, visual, acoustic and tactile contact are available and should be used in preference where animal isolation is complete.

##### Vascular access ports

Surgically implanted vascular access ports (VAPs) greatly facilitate repeated blood sample collection, blood pressure monitoring and infusions. Their use as a safe and efficient method for obtaining reliable chronic vascular access in farmed animal species has been described.^43-46^ Vascular access buttons achieve similar goals but obviate the need for (potentially painful) skin puncture.

##### Urine collection

Collecting free-flowing urine samples with a paper dish is facilitated by training and species-specific manoeuvres. For example, activating the nipple on drinkers can induce urine flow in recalcitrant pigs,^47^ while inducing transient apnoea promotes urination in sheep.

##### Cerebrospinal fluid sampling

Cerebrospinal fluid (CSF) sampling can be achieved on a single occasion by lumbar puncture in sedated animals^48^ or repeatedly using surgically implanted intrathecal catheters in the cisterna magna or cerebral ventricles.^49,50^

##### Behavioural studies

Digital photography and/or video recording provide data for behavioural studies in free-moving animals in familiar environments and are additionally useful for postoperative observation.^51^

### General (all species) recommendations

The following recommendations are applicable to laboratory pigs, sheep, goats and cattle.

● *Highly recommended:* Codes of recommendations for the welfare of livestock published by relevant bodies within the EU member states and the UK, and which support the national legislation protecting farmed animals should be used to define the minimum standards for handling and restraining farm animals under laboratory conditions. The *constant* refinement of methods for behavioural preparation and physical restraint should be prioritized.

● *Highly recommended:* All animals of all ages should undergo behavioural preparation in all studies in which these measures are likely to improve animal welfare and/or scientific outcomes. Out-sourced animals (from farms, other institutions, licensed breeders or livestock markets) should undergo behavioural preparation for a minimum of 14 days before study enrolment, except in specific circumstances (see non-recovery procedures). All animals should be habituated to acceptable methods of physical restraint.

● *Highly recommended:* The methods of physical restraint applied, where necessary, must be the least-aversive commensurate with achieving procedural objectives. Anxiolytic-sedative drugs should be used when physical restraint alone is stressful. Local and/or general anaesthetics with or without analgesics must be used when procedures are noxious.

● *Highly recommended:* Positive reinforcement methods should be used to facilitate non-invasive or mildly invasive procedures, e.g. physical examination, ultrasound and blood sampling.

● *Highly recommended:* Mixing unfamiliar animals must be avoided.

● *Highly recommended:* The young of all species should remain with their dams until behavioural (sexual) or scientific reasons justify separation. Where infeasible, compliance with best practice regarding weaning and/or specific regulations must be met. In some cases, e.g. piglets, specific exemptions must be sought and approved.

● *Highly recommended:* Animals must not be isolated except when isolation is an experimental necessity and has received regulatory approval. When animals require individual housing as an experimental necessity, or for reasons of animal safety, e.g. to prevent pen-mate interference with surgical wounds or catheters, or undesirable sexual interaction, separated animals should have visual, olfactory, auditory and limited tactile access to conspecifics, ideally with previous pen-mates. Metabolism cages that require individual housing but allow for full sensory contact should always be used in preference to complete animal isolation unless study objectives are undermined.

● *Highly recommended:* Food animals for scientific purposes should not be obtained from public markets or similar unless details of their life-history are known.

● *Recommended:* Food animals for scientific purposes should be bred and reared ‘on-site’ under similar conditions to those in which the experiments will be conducted.

● *Recommended:* Animals which cannot be bred on-site must be sourced from farms or other institutions with the highest welfare standards and where conditions are the least dissimilar from the destination laboratory. Ideally, such farms should be closely located to minimize transport times and stress.

### Specific considerations

The species-specific recommendations made in this section, which are based on species-specific considerations, are to be used in conjunction with the general recommendations made previously.

#### Pigs: considerations

##### Behavioural preparation

Pigs are intelligent and have advanced problem-solving abilities so are good candidates for behavioural preparation. They learn from their environment and readily adapt to new situations. They can manipulate objects to obtain food or solve challenges; cognitive stimulation is important for their well-being.^52^ Training should be initiated during the acclimatization period and continued throughout project-specific treatments for the study’s duration.^26,36,50^ A two-week, four-step habituation and socialization process facilitating urine and blood sampling (via implanted cannulae) and abdominal ultrasonography has been reported.^47^

While behavioural preparation is facilitated by optimized environmental conditions, the difference between basic animal needs and environmental enrichment must be recognized. Inadequate bedding can cause hyperactivity, aggression, excessive use of drinkers and the repetitive banging of pen panels; adequate bedding is, therefore, a necessity.^53^ Deep bedding satisfies the pig’s drive to burrow (root), prevents stress-related stereotypic behaviours and facilitates handling, but does not necessarily constitute enrichment.^54^ Formalized methods of scoring the progress of acclimatization, familiarization, habituation, socialization and training have been described in pigs.^55^

##### Isolation

Pigs with procedural *accoutrement*, e.g. external cannulae and stomata, will attract destructive investigation by pen-mates, so must be separated. Under these circumstances, animals should be habituated to both implants and separation beforehand. When individual housing is required, it is important to ensure that visual, olfactory, auditory and limited tactile access to conspecifics is allowed. This may be achieved using bar-separated pens, ‘snout holes’ in (ideally) transparent, i.e. Perspex walls and/or periodic supervised access to walkways allowing ‘visits’ to adjacent occupied pens. Regular and frequent rewarding human contact can serve as a substitute for social housing.^34,39^ Pleasant interactions, such as positive reinforcement, e.g. patting, scratching, rubbing and offering high-quality food rewards, should be implemented during this period. Environmental enrichment stimulating exploratory behaviours, i.e. new objects, manipulation (prehensible ‘toys’) and burrowing (deep straw) must be present in ‘separation’ pens.

##### Physical restraint

Pigs can resist physical restraint energetically although this depends on the method and the animal’s age, breed and training.^56^ Pigs can remember human behaviour in terms of negative^57^ or positive events.^58^ This influences their reactions to humans and so affects their welfare. Older, habituated pigs react less violently than younger, less well-handled animals. Unsympathetic physical restraint has been reported to cause traumatic liver lesions in neonatal Göttingen minipigs.^38,39,59^ Pigs communicate through different vocal and olfactory signals and body language. Grunting, squealing and other vocal expressions convey emotions and intentions. It is highly recommended that these are taken to guide the proper use of restraint methods in pigs.

Common physical restraint methods in pigs, e.g. ‘snares’, ‘snitches‘ or ‘snatches’, should only be used when absolutely necessary because they may cause greater pain than the scheduled procedure as well as acute and chronic stress.^19,39,60,61^ The person snaring should be trained and competent and the snare should be purpose-designed. Enforced recumbency in ‘V-troughs’ is similarly condemned.

Limiting movement in laboratory pigs for innocuous, very brief procedures, e.g. rectal thermometry and intramuscular injections, is best achieved using familiar devices, e.g. weighing crates or (for penned animals) pig (driving) boards with or without positive reinforcement (offered food also provides a useful distraction). Pigs can be distracted with ‘forking’: firmly scraping the animal’s back with a fork or a back scratcher elicits pleasure responses.

For similar procedures in animals <10 kg, capture and brief suspension by the pelvic limb followed rapidly by thoracic support allows the horizontal animal to be wrapped in towel and embraced firmly with the lower jaw supported, i.e. as for dogs.^62,63^ However, the duration of the suspension stage must be minimized: it places the rectus abdominis muscle under tension promoting umbilical herniation.^64^ Göttingen minipigs weighing 8 kg have been habituated successfully to slings for blood sampling.^65^

Larger pigs can be restrained in proprietary purpose-built slings^34,38,39,63^ or crushes^66^ although device selection is important: poorly designed or ‘generic’ devices have caused epistaxis, bleeding gums and bruising and must be avoided in pigs with bleeding disorders.^67^ Complications are less likely with equipment purposefully designed for immobilization and procedures such as blood sampling, physical examination and substance administration (intravenous and oral).^39^ Devices for the humane immobilization of pigs should support the head and neck, be of robust construction and be readily cleaned and disinfected. Slings must allow access to specific body areas when physiological instrumentation is *in situ*, for special procedures such as abdominal ultrasonography, blood sampling or the administration of medications. The design must consider the animal’s height, length and weight, its limbs, the external genitalia of male animals and the animal’s need to urinate and defaecate. It is highly recommended that pharmacological restraint be used for noxious procedures in pigs of all ages.

##### Urine collection

Foley catheters placed under deep sedation can be used for repeated urine sampling in female pigs, but the pig must be kept in an individual cage until the end of the sampling period to avoid interference from other pigs.^68^

#### Pigs: recommendations

##### Behavioural preparation

● *Highly recommended:* Mixing unfamiliar pigs disrupts social hierarchies and results in post-mixing aggression. If mixing is unavoidable, it should be phased and involve pigs of minimal age and size differential as soon as possible following legal weaning times, i.e. 3 weeks. It should be carried out early morning allowing trained personnel to be present (and to intervene) during the highest risk period or in the evening (before lighting is discontinued). Mixing pens should be large, contain visual barriers (allowing escape space for subordinate animals) and enriched, i.e. toys and straw. The use of olfactory distractants and/or anxiolytic drugs, e.g. azaperone or acepromazine, may be considered.

##### Physical restraint

● *Highly recommended:* The use of snitches (‘snares’ or ‘snatches’), V-troughs, ear suspension or prolonged limb suspension (not >5 seconds) must not be used in laboratory pigs.

● *Highly recommended:* Limiting movement in laboratory pigs for innocuous, brief procedures, should be achieved using familiar devices, e.g. weighing crates or pig boards with or without positive reinforcement.

● *Recommended:* Pigs should be habituated to slinging if these are to be used post-procedurally. Slings for heavier pigs should be customized to meet individual pig and procedural requirements.

● *Recommended:* Younger (smaller) animals and minipigs should be wrapped in towels and embraced, with the lower jaw supported, or held firmly in a manner like that in dogs.

##### Pharmacological restraint

● *Highly recommended:* Pharmacological restraint should be used for noxious procedures in pigs of all ages. Pigs should be sedated for all potentially stressful non-invasive, e.g. radiography (in untrained, non-standing animals), or minimally invasive, e.g. venous cannulation, procedures.

#### Sheep: considerations

##### Behavioural preparation

Being flock animals, sheep have a strong instinct to form cohesive groups, which provides a sense of security, and under natural conditions, aids in protection against predators. Flock behaviours, i.e. a tendency to follow a leading animal or flee *en masse*, are seen in groups of four or more animals, and account for sheep becoming distressed when isolated or separated from companions, although this depends on breed and age.^69–74^

Minimizing stress in laboratory sheep requires that aversive procedures are avoided until animals are acclimatized.^72^ Animals obtained from commercial, extensively husbanded flocks in particular require habituation to human contact and handling.^19^ Laboratory sheep benefit from environmental enrichment which promotes the diversity of natural behaviours while reducing stereotypies and aggressive behaviours.^27^ The strong escape proclivity of sheep can be reduced by space restriction, although this itself requires habituation.^75^

Enforced separation from the flock or familiar conspecifics is ameliorated by the presence of at least one other familiar animal. The effects of repeated blood sampling on haematological variables in sheep, which are well-recognized^76^ are reduced by providing *ad libitum* food and water and the presence of a social partner.^18,77^

Sheep are readily trained: positive anticipation training using food rewards to conditioning behaviour is particularly useful.^78^ Sheep can be trained to voluntarily enter a restraint device when they can move as a group and procedures are repeatedly imposed.^35,72^ Positive events, e.g. handling or wool brushing reduces wariness of humans, although this effect may not be transferable to all situations.^79^

Reviews of methods assessing behavioural reactions to handling and restraint in sheep^18,26,72^ generally affirm that the degree and signs of evoked fear depend on how the animal perceives handling (or transport).^19^ This may be useful in quantifying the success of acclimatization before animals are enrolled in experiments. An interaction between oxytocin release, sheep–human bonding and stress responses has been identified.^80,81^ This supports the pre-experimental acclimatization of sheep.^18^

##### Physical restraint

Under farm conditions, many potentially aversive procedures, e.g. shearing, ‘drenching’ or foot trimming, are performed with the animal restrained manually by being forcibly positioned upon its *tuber ischii* or ‘pin-bones’. Less commonly, restraining booths may be used. These and other procedures, e.g. dipping, are less aversive when conducted briskly and *en masse*.^82^

In certain circumstances, minor procedures can be conducted in standing sheep with minimal physical restraint and without drugs. Accessing intrathecal catheters at the lumbar reservoir or the *cisterna magna* for CSF sampling is possible with mild physical restraint alone.^77,83^ Access to implanted sampling devices is also facilitated in non-sedated animals when they are confined in a familiar, but size-restricted pen.^84^

Most sheep are accustomed to enforced restraint upon their ‘pin-bones’. Consequently, injections can be made, and blood samples taken without sedation in animals accustomed to and restrained in this way.^85^

Sheep may be restrained in canvas slings, the height of which allow the hooves to be in contact with or suspended above the floor.^77^ Slings may be useful postoperatively in supporting recovery from painful or prolonged surgery as they facilitate breathing and intestinal movement until the animal is able to stand unaided. Habituation to slinging is advisable to avoid adverse reactions, e.g. anxiety and struggling.^86,87^

Commercial sheep chairs are available and make non-invasive imaging techniques, e.g. ultrasonography, possible without sedation^82,88^ although the results may be confounded by the accompanying tachycardia, tachypnoea, vocalization and/or regurgitation.^82,89,90^ Refined methods for cardiac ultrasonography in standing sheep have been described and suggest an abducted limb is more acceptable than standing with the thoracic limb extended.^88^ Restraining booths may be used in laboratory sheep, but care is necessary when the risk of accidental de-instrumentation exists.

Introduced in the 1980s, proprietary electro-immobilization devices proved to be more aversive in sheep than physical restraint.^18^ They have no place in laboratory animal restraint, and their use is banned in the UK and EU member states.

##### Telemetry

Telemetric monitoring of foetal haemodynamic variables in pregnant sheep allows unrestricted movement in pens equipped with several telemetry receivers.^45^

Similarly, respiration chambers allow free animal movement during experiments of several days’ duration.

##### Metabolism cages

The combination of respiration chambers with metabolism cages allows sampling without the need for further restraint although prior acclimatization is required^91–94^ and absolute isolation is condemned.

#### Sheep: recommendations

##### Behavioural preparation

● *Recommended:* Heavily fleeced sheep should be sheared to reduce risk of inexpert physical restraint (see below) and heat stress and to improve clinical observation, e.g. breathing rate and pattern.

##### Physical restraint

● *Highly recommended:* Sheep may be physically restrained in a standing position or on their *tuber ischii*. Sheep must not be restrained by holding and/or pulling the fleece.

● *Recommended:* Physical restraint with local anaesthesia is suitable for blood sampling and short-term venous cannula placement.

##### Miscellaneous

● *Highly recommended:* Heavily fleeced animals should be sheered on admission to the facility if they are to be housed indoors.

#### Cattle and calves: considerations

##### Behavioural preparation

Most *bovidae* involved in research are young, i.e. calves. In these, separation from the dam, and later, conspecifics are major psychological stressors.^19^ Early weaning and individual housing have profound effects on the calf’s physical and psychological development.^95^ Environmental enrichment of individual housing has minor or insignificant benefits for calves, compared with the presence of other calves.^96^ Paired housing systems promote greater daily feed intake and if calves are joined in pairs from birth, rather than a few days later, they exhibit fewer behavioural disruptions caused by early weaning.^97^

Calves are easier to handle and become less stressed if habituated and trained, i.e. handling occurs during acclimatization. Calves accustomed to handling have lower cortisol levels after restraint than those that have less-frequent human contact.^98^ Younger, bottle-fed calves acquired from the dairy herd at 2–2.5 months of age are easier to habituate than older calves.^99^

##### Physical restraint

Physical restraint of the standing calf, with or without halters or manual head restraint, is appropriate in adequately acclimatized and habituated calves for minor, brief non-invasive procedures such as physical examination, postoperative wound care, blood sampling from permanent central venous cannulae, etc. For more invasive procedures, the use of local or systemic analgesia decreases acute pain and is strongly recommended.

Commercially available devices are available for prolonged restraint. One^100^ involves a mobile calf pen with a hydraulic lifting device. In this, calves wear a sling of reinforced nylon webbing with padded openings for the extremities and an adjustable central opening to prevent pressure on the abdomen. The pen is designed so that calves can stand or lie down at will or be restrained in an upright position when necessary.

Older male *bovidae* are seldom involved in research but require particular care when they are. They often develop aggressive tendencies, especially if entire, and despite behavioural preparation. Experienced staff and purpose-built handling facilities are necessary. More robust restraint methods, i.e. crushes will be required in these to reduce the risk to personnel conducting even innocuous procedures.

While young cattle are best housed in groups their play and exploratory behaviours may result in displacement of unprotected external instrumentation, e.g. central venous cannulae.

##### Telemetry

Non-contact monitoring does not affect cattle behaviour or induce stress, so is particularly useful for diagnosing respiratory disease.^101^ Telemetry has been used in calves in hemodynamically complex diseases such as pulmonary arterial hypertension.^102^

#### Cattle and calves: recommendations

##### Behavioural preparation

● *Highly recommended:* Growing calves should be habituated to physical restraint. Adult cattle should be habituated to head-yolks and ‘crushes’.

##### Physical restraint

● *Highly recommended:* Calves should be restrained in the standing position for pain-free procedures such as clinical examination. Adult cattle should be restrained using head-yolks or ‘crushes’. In *bovidae* of all ages, physical restraint with local anaesthesia should be used for vascular port sampling, blood sampling and short-term venous cannula placement.

#### Goats: considerations

##### Behavioural preparation

Goats are renowned for their exploratory and climbing ability (with the latter reflecting the former) arising from their tendency to forage rather than graze. Consequently, goats are regarded as more curious, quicker-to-learn^103^ and easier to handle than sheep and so are easier to acclimatize although their behaviour may be less predictable.^104,105^ Laboratory goats are usually group-housed under environmentally enriched conditions^106^ which promotes their learning performance and confers other behavioural benefits.^107^ Mixing unfamiliar goats can promote stress and impair welfare for up to 5 days.^108^ Horned animals require special attention because of the risk of injuries.^108^ Visual, auditory and tactile contact with group members should be maintained when goats are physically separated from pen-mates in order to facilitate reintroduction.^109^ The behaviour and well-being of goats depends on their relationship with animal care technicians and upon previous experiences with human beings.^105,110,111^ Goats readily habituate to experimental conditions, e.g. the wearing of belts for body function measurements^103,112^ although learning experimental procedures in a group, or introducing an experimental setup into the animals’ familiar environment can limit anxiety and excitement.^112^

##### Physical restraint

Restraint methods commonly used in sheep, e.g. physical restraint by hand, harness or holster, are suitable for standing goats.^113^ In studies in which human presence is ideally minimal, goats are restrained when necessary by familiar handlers.^114^ Some studies, e.g. respiratory gas measurements, can be completed without restraint although animals should be acclimatized to measurement conditions for 2–3 days beforehand.^114^ Cardiac ultrasonography can be conducted on unsedated standing animals after habituation to the experimental conditions.^115^ Access to previously implanted sampling devices, e.g. intrathecal cannulae, is possible in unsedated standing goats when repeated sampling of CSF fluid is required.^113^ Slings can be used to facilitate husbandry procedures such as hoof trimming. The successful use of a sling is reported in a study on hip arthroplasty when the sling was used for preventing goats from lying down.^116^

##### Telemetry

Implantable telemetry devices allowed the 24-hour recording of heart rate and blood pressure in the same unrestrained animals and to identify the effects of routine manipulations and reproductive cycle.^117^

#### Goats: specific recommendations

##### Behavioural preparation

See all species (above).

##### Physical restraint

● *Highly recommended:* Goats can be restrained in the standing position for examination. Physical restraint with local anaesthetics should be used for blood sampling and short-term venous cannula placement.

### Conclusions

Optimizing the welfare of farmed animal species which have not been reared under laboratory-type conditions can be mitigated by careful source selection, appropriate habituation and familiarization which will further improve the animal’s preparedness for study. While time-consuming and costly, its benefits in terms of improved welfare, experimental refinement and data quality are likely to be substantial. Whenever animals cannot be trained to accept scientific procedures, the judicious combination of physical and pharmacological restraint will be required, and their attendant effects on scientific outcomes considered.

## Part II: General principles of anaesthesia for pigs, sheep, goats and cattle involved in biomedical research

### Introduction

Russell and Burch believed that anaesthesia was the most important component of experimental refinement.^2^ However, subsequent and ongoing developments in veterinary anaesthesia and analgesia have made it increasingly difficult to prescribe, i.e. make *specific* recommendations, for anaesthetics and analgesics in large animals involved in biomedical research. Numerous factors, many of which are poorly characterised, unpredictable, immeasurable or even unidentified, conspire to ensure that a technique proving ideal in one experiment may be found wanting when the same study is conducted elsewhere (which undermines the principles of animal reduction and experimental reproducibility). Focussing on anaesthetics rather than analgesics, and on pigs, sheep, cattle and goats undergoing procedures requiring general anaesthesia this section will; (i) demonstrate the importance of anaesthetic technique development based on specific project details; and then (ii) make recommendations intending to limit problems with anaesthetic mismanagement. Laboratory animal anaesthetics should: (i) *refine*, i.e. provide conditions that allow potentially unpleasant procedures to be conducted with the least (ideally, no) adverse physical or psychological consequences for the animal; and (ii) *reduce*, i.e. allow the collection of enough data of sufficient quality to confirm (or reject) scientific hypotheses using the fewest animals possible. The role of adequately trained personnel in planning and managing anaesthesia and analgesia is pivotal to minimizing the effect of experimental procedures on animals.^17^

### Anaesthesia in large laboratory animals: challenges

The challenges in providing anaesthesia and analgesia to large animals for experimental procedures are different to those conducted for commercial purposes under farm conditions. The anaesthetic and analgesic techniques employed under the latter conditions are seldom appropriate in the former because: (i) scientific procedures differ markedly from those conducted on production animals; some being complex, prolonged, unfamiliar to the operator and/or especially noxious; (ii) research animals are less likely to enter the food chain; and when this is the case, adherence to *some* prescription-related regulations is unnecessary (specifically, regulations controlling the use of drugs affecting meat or milk quality or threaten consumer health can, unlike the case in commercial animals, usually be used in laboratory animals not destined for consumption; regulations pertaining to prescribing authority and the use of controlled substances remain applicable); (iii) there is a moral obligation to ensure experimental refinement, i.e. minimizing the effect of the procedures on the animal; while (iv) similarly ensuring, wherever possible, that scientific outcomes are achieved.

Anaesthetic and analgesic drugs (and pain) are potentially major confounders of scientific data. Consequently, details of anaesthetic and analgesic techniques and other features of periprocedural care should always be reported within or supplemental to all publications involving animal research. Details of new and/or refined techniques should be reported as standalone publications in journals likely to be read by those responsible for animal care and welfare. This recommendation is in accordance with the first (2010) and the second (2020) ARRIVE guidelines^15,118^ which specify the details to be documented: (i) the approach to animal preparation for anaesthesia and procedures; (ii) details of animal husbandry; (iii) anaesthetic and analgesic drug doses; (iv) routes and frequency of administration; (v) animal monitoring; (vi) pain assessment before, during and after the procedure; (vii) the fate of the animals at the experiment’s end; and (viii) the incidence of adverse or unexpected events.^15,118^

### Anaesthesia: fundamental concepts and definitions

Understanding fundamental concepts of anaesthesia and analgesia are prerequisites to the development and performance of safe and effective periprocedural practices.

Anaesthesia is the controlled and reversible elimination of sensation achieved through the precise administration of anaesthetics. The process involves the modulation of neural pathways at various levels, leading to the suppression of conscious awareness and memory, attenuation of noxious stimuli perception, and loss of muscle tone, thereby facilitating procedures with minimal subject discomfort. Balanced anaesthesia is the combined use of drugs selected to produce unconsciousness and amnesia, muscle relaxation and analgesia.^119^ The management of pain resulting from noxious interventions is incorporated into balanced anaesthetic techniques as part of a multimodal and pre-emptive analgesia strategy which may be continued into the postoperative period.^119^ Multimodal analgesia involves the administration of analgesic drugs with different modes of action.^120^ Preventive analgesia aims to prevent peripheral and central pain sensitization and relieve pain beyond the expected duration of drug action.^121^

While general anaesthesia immobilizes animals and allows procedures to be accomplished humanely, it is not always necessary: some procedures can be completed using physical restraint and/or sedation with appropriate analgesia or local anaesthesia.

Sedation is a state of reduced sensibility, awareness and reflex obtundation from which animals are arousable. Some non-invasive procedures can be completed in sedated animals but unresponsiveness to certain stimuli,^122,123^ including pain, cannot be guaranteed. Neither can immobility: noxious procedures cannot usually be performed on sedated animals without local or regional anaesthesia.^123^ Local anaesthetics are extremely effective because an appropriate administration technique and dose ensures complete sensory (and usually motor) blockade.^123^ Local anaesthetics can be administered in numerous ways either alone or in conjunction with sedation or anaesthesia.^124^ Local anaesthetic techniques are well-described in pigs, sheep, goats and cattle: see Hall, Clarke and Trim’s *Veterinary Anaesthesia*.^125-127^

Acclimatization and habituation of animals to personnel, equipment, interventions, the environment and experimental conditions, along with training, may preclude the need for sedation or anaesthesia altogether, particularly when the procedures involved are brief and painless. The time and effort spent on training is most worthwhile for long-term studies involving repeated non-painful procedures.

The advantages and disadvantages of general anaesthesia, sedation and local anaesthesia vary according to the procedure to be performed, animal factors, the skills and experience of personnel and the availability of equipment.

In human subjects, the nature, quality, severity and location of postoperative pain is broadly related to the operation performed. Consequently, procedure-dependent approaches to anaesthesia and analgesia have recognized practical value.^128-130^ Guidelines for species-specific considerations in pain management (assessment, quantification and treatment) are available for rodents and rabbits.^131^ These associate estimated pain levels with procedures of increasing invasiveness, i.e. from minimal–mild, e.g. ear notching and superficial tumour implantation, through mild–moderate, e.g. orchiectomy and embryo transfer, to moderate–severe, e.g. laparotomy and thoracotomy.^131^ An estimate of pain severity associated with specific procedures can similarly be made for pigs, sheep, cattle and goats adjusted for other factors affecting their pain experience. In particular, the anatomical location, invasiveness and duration of surgery, the extent of tissue trauma (frequently related to the surgeon’s experience and skills), the animal’s health (both physical and psychological), the anaesthetic and analgesic technique selected and the severity of surgical complications should be considered (Tables 1 and 2). The quality of postoperative (analgesia and supportive) care is of major importance, which means these considerations apply less to procedures performed under terminal anaesthesia.

**Table 1.** Estimated pain severity (none–mild–moderate) associated with experimental procedures in production animals. Estimates based on the anticipated degree of nocistimulation experienced and the necessity for extensive, aggressive and considered peri-procedural analgesic therapy.

Estimated pain	Procedure	Species-based examples			
		Pigs	Sheep	Cattle	Goats
None	Imaging	Cardiac MRI^132^	MRI: fetal hepatic blood flow^133^		MRI (limbs and brain)^134-136^
	Clinical examination			Immunological studies^137^ Appetite studies^138^	
Minimal to mild	Ocular surgery	Robotic surgery training^139^		Corneal transplantation^140^	
	Cardiovascular catheterization	Coronary device evaluation^141^	Ventricular catheterization^142^		
	Dental surgery	Dental implant testing^143^	Dental implant testing^144^ Mandibular osteogenesis^145^		
	Head and neck (soft tissue) surgery	Thyroid surgery^146^			
	Endoscopy	Bronchoscopy^147^	Lung denervation^148^	Bronchoscopy and endobronchial inoculation^149^	
Mild to moderate	Minor laparotomy		Ovariectomy^150^		Ovariectomy^151^
	Dental surgery	Dental implant testing and mandibular osteogenesis^152^			
	Minimally invasive surgery	Laparoscopy (training)^153^ Thoracoscopy^154^	Laparoscopic uterine surgery^155^	Laparoscopic renal surgery (nephrectomy)^156^ Laparoscopic embryo transfer^157^	Cardiac pacemaker implantation^158^ Embryo transfer^159^
	Craniotomy	CSF leak repair^160^	Cranial flap fixation^161^ Skull base reconstruction^162^		

**Table 2.** Estimated pain severity (moderate–severe) associated with experimental procedures in production animals. Estimates based on the anticipated degree of nocistimulation experienced and the necessity for extensive, aggressive and considered peri-procedural analgesic therapy.

Estimated pain	Procedure or model	Species-based examples			
		Pigs	Sheep	Cattle	Goats
Moderate to severe	Orthopaedic surgery	Tibial implant^163^	Osteotomy^164^	Cuff rotator surgery^165^	Bone augmentation^166^ Bone-defect repairs^167^ Ligaments damage^168^ Cartilage damage^169^ Arthrotomy^170^
	Major laparotomy	Bariatric surgery^171^ Liver transplantation^172^ Kidney transplantation^173^ Intestinal transplantation^174^ Cholecystectomy^175^ Pancreatic ablation^176^ Foetal cell injection^177^ Sepsis induction^178^	Foetal surgery after maternal laparotomy and hysterotomy^179,180^		
	Thoracotomy	Lung resection^154^ Myocardial infarction^181^ Oesophageal anastomosis^182^	Myocardial infarction (coronary ligation)^183^	Artificial heart implantation^184^	Myocardial infarction^185^ Pneumonectomy and xenotransplantation^186^
	Sternotomy	Mitral valve replacement^187^	Cardiopulmonary bypass^188^		
	Maxillofacial surgery	Bone Regeneration^189^			
	Models of painful conditions in humans	Pancreatitis^190^ Burns^191^ Trauma^192,193^ Reproductive diseases^194^		Reproductive diseases^194-196^ Polycystic ovarian syndrome^197^	Osteochondrosis^135,136^ Osteoporosis^198,199^

### Anaesthetic technique recommendations: pitfalls

Anaesthesia for animals in experimental procedures can be produced in numerous ways because the wide range of anaesthetic and analgesic drugs available exponentiates the number of possible combinations arising therefrom. The dose range of individual agents producing clinical effects, different routes of administration, and the use of ‘adjunct’ drugs, e.g. inotropes, antimuscarinic drugs, anticonvulsants and antiemetics, to provide physiologic support or to control adverse phenomena caused by surgery, further extends the range of suitable anaesthetic techniques. Importantly, anaesthesia must not be regarded as a set of instructions in which specified drugs are given in a set sequence at predetermined doses by prescribed routes. Other, non-pharmacological considerations complicate the formulation of an anaesthetic technique and may indeed have a greater effect on study outcome than the drugs used (Table 3; column 3).

**Table 3.** Pharmacological and non-pharmacological elements needed to achieve the objectives of the six periods of an anaesthetic technique.

Anaesthetic Period	Objective(s)	Non-pharmacological elements.	Pharmacological elements (select drugs, doses, routes of administration)
Preoperative examination	(i) Identify risks associated with procedures and anaesthesia. (ii) Provide information for preoperative preparation and anaesthetic technique selection.	(i) Review medical and previous procedural history. (ii) Physical examination. (iii) Further examination, e.g. haematology, biochemistry, imaging and electrocardiography, if necessary.	
Preoperative preparation	Reduce risks associated with the scheduled anaesthetic.	(i) Ensure availability and adequacy of personnel, equipment and facilities. (ii) Select anaesthetic technique. (iii) Determine body mass. (iv) Consider preoperative food and water deprivation.	
Pre-anaesthetic medication	Administer drugs reducing anaesthetic doses.	Consider pre-induction oxygenation; physiological monitoring.	Sedatives Anxiolytics
	Administer drugs supporting procedure.	Practical objective of pre-anaesthetic medication is physical restraint	Analgesics Antibiotics Antimuscarinics Anti-inflammatories Antiemetics
	Facilitate venous cannulation.		Sedatives Anxiolytics
Induction of anaesthesia	Produce unconsciousness.	(i) Select administration route, e.g. intramuscular, intravenous or mask. (ii) Select vein and cannulation technique. Practical objective: to provide conditions for tracheal intubation.	Induction agent(s)
Maintenance of anaesthesia	Produce stable conditions for the procedure and safety for animal and operators	(i) Select airway management, e.g. tracheal tube, laryngeal mask, tracheostomy or mask. (ii) Select breathing system, e.g. rebreathing versus non-rebreathing. (iii) Select ventilatory mode, e.g. spontaneous, assisted or controlled. (iv) Consider body positioning and implications. (v) Consider fluid volume replacement. (vi) Consider urinary bladder catheterization and urine collection. (vii) Consider ocular and integumentary protection. (viii) Select methods to support thermoregulation. (ix) Select physiological variables for monitoring.	Anaesthetics Analgesics Muscle relaxants Fluids Local anaesthetics Adjunct drugs
Recovery from anaesthesia	Accelerate restoration of pre-procedural state	(i) Elimination and antagonism of anaesthetic and adjunct drugs. (ii) Pain management. (iii) Physiological monitoring with restoration of adequate ventilation, tissue perfusion, circulating volume and core temperature. (iv) Staged withdrawal of non-vital physiological support. (v) Wound management. (vi) Body repositioning. (vii) Restoring eating, drinking and thermoregulatory behaviours. (viii) Restoring eliminative, grooming and kinetic behaviours.	Analgesics Sedatives Adjunct drugs Drug antagonists

Establishing a suitable anaesthetic technique requires consideration of: (i) the target species; (ii) individual animal characteristics; (iii) the anticipated procedure(s); (iv) human factors; (v) equipment factors; and (vi) the experiment.

#### Species characteristics

Differences in the biological characteristics of small ruminants, pigs and calves affect non-pharmacological elements of anaesthesia more than drug selection. An anaesthetic must accommodate the target species with respect to their: (a) anatomical; (b) ethological; (c) physiological; and (d) pharmacological characteristics. This point is illustrated by providing examples, rather than a comprehensive review.

##### Anatomy

The superficiality of jugular veins makes central vein cannulation straightforward in unsedated calves and sheep compared with pigs. Differences in lumbosacral spinal cord anatomy will affect techniques for extradural and spinal injection in farmed animal species. The presence of a right accessory bronchus in pigs demands attention to tracheal tube length.

##### Ethology

Pigs react to physical restraint more violently than small ruminants and calves, which justifies the use of intramuscular sedative drugs before attempted venepuncture.

##### Physiology

The functional rumen which develops in growing calves, lambs and kids creates problems, e.g., ptyalism, tympany, regurgitation-aspiration, that are seldom encountered in monogastric species. Preoperative food and water deprivation requires greater consideration in ruminants than in suidae.

##### Pharmacology

Inter-species differences in drug sensitivity and dosing are drug-dependent. The minimum alveolar concentration (MAC) of volatile anaesthetics varies marginally when compared with responses to α_2_ agonists.^200^ There are also absolute differences: paracetamol causes methaemo-globinaemia in pigs, but not sheep.^201^ There are major differences between sheep and pigs in their response to neuromuscular blocking agents (NMBAs).^202^

#### Individual characteristics

Intra-species variation in the response to anaesthetics may be intrinsic, i.e. pharmacogenomic, and unquantifiable, that is until the anaesthetic is given and the effects are observed.^203^ Other widely recognized factors affecting drug responses and technique selection include age, sex, temperament, size, breed, health status, concurrent medication, reproductive status and source.

##### Age

Very young or aged animals are generally believed to be more sensitive to fixed drug doses because of a reduced clearance capacity. Age-related changes in body composition introduce similar pharmacokinetic variability. The central nervous effects of anaesthetics are age-dependent in other species.^204,205^ Anaesthetic equipment designed for adult human use which is suitable for size-relevant pig and sheep models may cause critical problems if used in minipigs, piglets or lambs.

##### Sex

Sex differences in drug biotransformation and pharmacokinetics have been identified in sheep^206^ and pigs^207^ but their relevance to anaesthesia is unknown. Catheterization of the urinary bladder via the urethra is not possible in male sheep nor male pigs, so direct bladder catheterization is required if urine output is to be measured.

##### Temperament

Animals acclimatized to the laboratory will generally be more sensitive to fixed anaesthetic doses compared with non-acclimatized animals.

##### Size

Allometric principles are well-recognized and applied in small laboratory animal species^208^ although their clinical relevance in farmed animals is less well-known. However, basic thermodynamic principles predict that heat loss during anaesthesia is an inverse function of body size so anaesthetics promoting this will have a disproportionately greater effect on body temperature in smaller individuals. Consequently, ambient (laboratory) temperatures are of greater importance when anaesthetizing lambs, piglets and minipigs compared with adult or larger animals.

##### Breed

Breeding affects temperament in sheep and pigs, but whether this extends to anaesthetic sensitivity is unknown. Ethnicity affects anaesthetic requirement in human beings^209^ while sheep breeds differ in their sensitivity to xylazine^210,211^ which may have a genetic basis. It seems possible that the inbred docility of some laboratory breeds, e.g. the Göttingen minipig, renders them more sensitive to sedatives than less well-handled commercial pig breeds. Breed predisposition of pigs to malignant hyperthermia, a condition triggered by stress, as well as specific anaesthetics, is a well-recognized pharmacogenetic problem in veterinary anaesthesia.

##### Health status

Unhealthy animals, including genetically modified forms with morbid phenotypes, are seldom involved in studies because the underlying pathology may confound study outcomes. When this is not the case, greater care is needed in anaesthetizing sick animals because anaesthetic risk is increased according to the nature and extent of the disease present.

##### Habitus

Extremely obese or lean animals should not be enrolled on study unless these states are induced as a deliberate study feature. The pharmacokinetic behaviour of anaesthetics may be affected by extremes of subject habitus and will have secondary effects on dosing and/or drug selection.

##### Medication

Seemingly innocuous medications, e.g. antibiotics, may adversely interact with anaesthetics and must be considered when techniques are selected.^212^

##### Pregnancy

The potentially abortifacient effects of α_2_ agonist drugs^213^ and glucocorticoids^214^ should be considered in studies involving pregnant animals. The use of non-steroidal anti-inflammatory drugs (NSAIDs) may also affect experimental results in pregnant animals.^215^

##### Source

Whether an animal was obtained from a commercial or institutional farm, a laboratory animal breeder, or was ‘home’ (laboratory) bred probably plays an understated role on the animal’s responses to anaesthetics. Laboratory-bred animals will be more familiar with the environment than animals from commercial sources and so less anxious, making them more sensitive to drugs with sedative properties.^6^

#### Procedural factors

Features of the procedure (duration, invasiveness, required precision, initial versus repeat, operative site, terminal versus recovery) for which an animal is anaesthetized has a major effect on technique selection because some procedures will require levels of analgesia, muscle relaxation or autonomic nervous areflexia that an anaesthetic alone cannot safely provide.

##### Duration

*Ceteris paribus*, brief, non-invasive procedures justify the use of both short-acting drugs and non-invasive, rapidly applied (though less accurate) methods of physiological monitoring. The exclusion of some support measures, e.g. intravenous fluids, may also be justified. In procedures of indeterminable duration such as certain types of imaging, drugs allowing rapid recovery despite prolonged administration should be chosen, e.g. volatile anaesthetics.

##### Invasiveness

The extent of procedural tissue damage is a determinant of anaesthetic risk, postoperative pain and severity level estimation. More invasive procedures justify more extensive physiological monitoring during anaesthesia and recovery, there being a greater need for awareness of nocistimulation during anaesthesia (and pain thereafter), haemorrhage and volume replacement, and thermoregulation. The actual tissue damage incurred during a procedure determines the frequency and degree of postoperative care required.

##### Precision

Unexpected movement and/or haemorrhage complicates precision surgery. Irregular breathing movements may be controlled with positive pressure (lung) ventilation (PPV). In general, and when permitted, NMBAs are used to prevent reflex movement and muscle tone.^216^ Measures taken to minimize haemorrhage in precise procedures conducted under surgical microscopy are based on surgical site positioning and in special circumstances, hypotensive anaesthetic techniques.

##### Repeats

Repeating surgery at the same site has two consequences for anaesthetic management: (i) scar tissue formation and (ii) peripheral and central pain sensitization. The presence of scar tissue and adhesions will complicate and prolong subsequent surgeries with corresponding demands on anaesthetic management. This, and the possibility that nociceptive pathways have been ‘sensitized’ during previous operations, will necessitate increasingly aggressive perioperative pain management on subsequent interventions. Repeatedly mismanaged anaesthetics and/or postprocedural pain will contribute to major cumulative changes in an animal’s extended welfare assessment grid.^10^

##### Operation site

The operation site affects anaesthetic management in specific and general ways. For example, thoracic procedures require PPV, while endocardial procedures will require ECG monitoring and the availability of anti-arrhythmic drugs. Visceral exposure during laparotomy and thoracotomy will predispose to hypothermia. Unintentional surgical damage to large blood vessels may necessitate rapid volume restoration while inadvertent nerve traction may initiate unexpected motor responses, e.g. movement or parasympathetic nervous activity in the case of direct vagal stimulation. Regarding analgesia, tissues vary in the level and type of sensory innervation which may explain why certain procedures are associated with a greater risk of developing chronic post-surgical pain than others.^217^ Certain body areas are amenable to complete desensitization using local anaesthetic techniques, e.g. the distal antebrachium is anaesthetized after brachial plexus block.

##### Procedure

The effects of procedure on the pharmacodynamic/pharmacokinetic (PK/PD) profiles of anaesthetics must be considered and modifications made as necessary. Normal doses of NMBAs that rely on renal or hepatic clearance may prove excessive after transplantation procedures, which reduce renal or hepatic function.

##### Non-recovery procedures

While adequate anaesthesia and intraoperative analgesia must always be prioritized the need to prepare for acute and/or chronic post-procedural pain is unnecessary in animals scheduled to be terminated before recovery occurs.

#### Human factors

In medical practice, anaesthetic problems are less likely when operators are skilled and experienced^218^ supporting the adage that, ‘the good surgeon deserves a good anaesthetist, who is indispensable for the bad surgeon’.^219^ Another adage (ascribed to Robert Smith^220^) asserts that, ‘There are no safe anaesthetics, there are no safe anaesthetic techniques, there are only safe anaesthetists’. Personal experience with any given anaesthetic technique in a specific procedure is an important safety factor; it is also axiomatic that when faced with high-risk and/or unfamiliar cases, anaesthetists should favour techniques with which they are most familiar, and not those which are more appropriate in theory. The subject of anaesthetic competency is examined in the following.

#### Equipment factors

Planning anaesthesia involves identifying the equipment, as well as the drugs required. Safe anaesthesia requires a range of equipment commensurate with procedural and, correspondingly, anaesthetic complexity. The equipment chosen need not necessarily be the most accurate nor technologically advanced: there are problems with over-instrumentation.^221^ Equipment can be categorized as that: (i) required for drug delivery; (ii) physiological monitoring (and data recording); and (iii) supporting physiological function (Table 4).

**Table 4.** Anaesthetic equipment ranked approximately according to necessity.

Anaesthetic delivery	Physiological monitoring	Physiological support
**Essential (***Highly recommended***) items**		
Hypodermic needles and syringes	Stethoscope	Insulating (‘space’) blankets and/or ‘heating’ pads
Intravenous cannulae	Oesophageal stethoscope	
Medical oxygen cylinders	Rectal thermometer	Warm air blower
Face masks	Pulse oximeter	Heat and moisture exchangers
Mouth gags		Drip stands (for IVFT)
Laryngoscopes		
Endotracheal tubes/stylets		
Anaesthetic breathing systems		
Anaesthetic machine		
Lidocaine applicator		
Syringe drivers		
Laryngeal masks		
Anaesthetic vaporizer		
Anaesthetic gas scavenging		
**Desirable (***Recommended***) items**		
Nerve locators	Capnograph	Infusion controllers
Neuraxial needles	Thermistor probe	Stomach tubes
	Respiratory gas analyser	Rumen trocars
	Oscillotonometer (NIBP)	Endobronchial catheters
	Invasive blood pressure monitor	Endobronchial suction
	Urinary bladder catheters	Mechanical lung ventilator
	Urine collection system	Self-inflating lung inflator
**Potentially useful (*Suggested*) items**		
Neuraxial cannulae	Biochemical analyser	DC defibrillator
Wound ‘soaker’ catheters	Microhaematocrit	Plasmapheresis equipment
Ultrasonography	Blood gas analyser	Cardiopulmonary bypass
	Depth of anaesthesia monitor	

IVFT, intravenous fluid therapy; NIBP, non-invasive blood pressure; DC, direct current.

#### Study considerations

The aforementioned factors apply when *any* animals are anaesthetized, and when laboratory animals require anaesthetics: (i) for interventions which are critical to the study’s programme of work but which will not affect study outcomes *per se*, e.g. pre-study imaging for animal screening and selection or hysterotomies for oocyte collection in *ex vivo* reproduction experiments; or (ii) for non-experimental veterinary procedures, e.g. for post-study treatment of procedural complications such as wound infections. Considerably greater challenges arise when planning anaesthetics for studies in which the anaesthetics and/or associated procedures are likely to affect study outcomes directly or indirectly. This occurs when data are collected during the anaesthetic maintenance period or in the immediate post-anaesthetic period, when significant procedural and/or anaesthetic drug effects are likely to persist. Under these circumstances, devising an anaesthetic plan must simultaneously involve consideration of the (real or putative) effects of anaesthesia on the experiment itself while recognizing the problems arising from a ‘standardized’ technique. In addition, anaesthetic challenges can arise from additional study demands, i.e. extraordinary treatments, recovery *versus* non-recovery studies, external validity, ‘batch’ (i.e. numerous animals being anesthetized at the same time; this is possible in laboratory rodents where suitable anaesthetic equipment is available; in large animals, the term refers to members of a study group being anesthetized in rapid succession, rather than simultaneously) and repeated (i.e. repeatedly anaesthetizing a single or several animals at pre-determined intervals as part of the study’s plan of work) anaesthetics.

#### Effects of anaesthesia on the experiment

Nociceptive ‘noise’ results from stimuli which in conscious animals are painful. Some anaesthetics have limited ability to prevent nociception, in which case analgesic drugs (or techniques) are required. This seems most likely in procedures involving skin burning, craniotomies, laparotomies, laparoscopies, orthopaedics and thoracotomies, which are regarded as severely painful in laboratory animals by the American College of Laboratory Animal Medicine (ACLAM) Analgesic Task Force.^131^ However, while analgesics will limit nocistimulation, they can themselves affect physiological variables of experimental interest, particularly when overdosed, i.e. when nociceptive stimulation is overestimated. Specific analgesics must not be used in experiments in which they directly affect study objectives, e.g. NSAIDs in inflammation models. That analgesic drugs can be categorized into at least 12 distinct groups based on neuro-pharmacological action means it is seldom necessary to withhold analgesics drugs altogether from experimental animals, except in experiments studying pain itself.^222^ The effect of analgesics on variables of scientific interest has been reviewed extensively.^223^ Pharmacological anaesthetic ‘noise’ results from anaesthetic drugs directly altering variables of experimental interest, e.g. isoflurane and cerebrocortical electrical activity, or ketamine in N-methyl-D-aspartate agonist and/or receptor studies. This potential problem is limited by appropriate drug and dose selection. Physiological anaesthetic ‘noise’ arises from adverse coincidental effects of anaesthesia, e.g. hypotension, hypothermia and nocistimulation, on scientific outcomes, and is caused by anaesthetic mismanagement. The two are often related, e.g. hypoventilation caused by volatile anaesthetic and opioid analgesic administration in spontaneously breathing animals will cause respiratory acidosis with widespread (though readily avoidable) physiological effects.

Delivering anaesthetics and analgesics in a way that ensures noxious experimental procedures (see Tables 1 and 2) do not cause pain, suffering and distress (and the effects of these on data quality) while avoiding overdose, and its undesirable consequences, i.e. prolonged recovery, post-procedural morbidity or even mortality (and a similar deprecation of data quality) requires skills commensurate with the complexity of the procedure. Not infrequently, there will be disagreement between researchers and animal anaesthetists on the most suitable anaesthetic and/or analgesic technique to be used. If contentious, the proposed anaesthetic must be agreed between scientists (to ensure compatibility with study objectives), supervising veterinarians (for authority), the assigned anaesthetist (for feasibility) and the institutional Animal Welfare and Ethical Review Body (AWERB; UK) or Animal Welfare Body (AWB; EU Member States) to ensure regulatory compliance).

#### ‘Standardized’ techniques

In many studies, the pharmacological and non-pharmacological features of the anaesthetic (Table 3) and pain management must be adapted if needed and then standardized to limit their confounding effects on study outcomes. This points to the benefits of a universally accepted anaesthetic–analgesic technique which could be applied for specified procedures conducted in established animal models: such standardization would allow the comparison and pooling of data from different groups, accelerate scientific progress and reduce animal use. However, this utopian proposal would mitigate against experimental refinement based on the improvement of anaesthetic techniques. Furthermore, universally accepted animal models are relatively uncommon in farmed animals compared with traditional laboratory species. Finally, standardised anaesthetic techniques ignore the biological variability inherent amongst individuals and preclude ‘individualized medicine’: a recognized safety factor in anaesthetic practice.^224^

#### Extraordinary treatments

The effects (rather than the demands) of the experiment on anaesthetic technique selection must be considered. Some procedures directly (and considerably) increase anaesthetic risk and cannot be fully managed because they are an integral component of the study itself. Acute severe haemorrhage in trauma studies, hypoxia in neonatal cerebral protection studies and cardiac arrest in resuscitation studies exemplify this challenge. Other interventions are less challenging but nevertheless increase demands on the anaesthetists’ skills, e.g. heparin administration before vascular implant studies.

The study of unevaluated drugs, substances, devices or surgical procedures in anaesthetized animals may unexpectedly complicate anaesthetic management because the effects of the unevaluated are, by definition, unknown. Unforeseen events may also occur when genetically modified animals with incompletely characterized phenotypes are anaesthetized. In both examples, problem control should involve: (i) conducting cadaveric and/or pilot studies (initially under terminal anaesthesia); (ii) extending the range of physiological variables monitored; and (iii) being adequately prepared to manage life-threatening complications. When unevaluated surgical procedures are scheduled, measures should be taken to ensure that inestimable postoperative pain levels can be managed effectively.

The greatest challenge in anaesthetizing animals for ‘first-time’ interventions is that the personnel involved will have limited previous experience. Cadaveric pilot studies may partly resolve this problem for surgeons facing novel surgical procedures, but not anaesthetists. This challenge also raises a legal conundrum with respect to the anaesthetist’s competencies (discussed in the following).

#### Recovery and non-recovery studies

Factors affecting the rate and quality of recovery from anaesthesia are of limited importance in non-recovery experiments. This is not the case when non-recovery procedures are part of a staged programme of work leading ultimately to recovery experiments.

#### External validity

In studies in which animals are used to model human conditions, the preservation of experimental validity imposes a scientific and ethical imperative that the model be treated, as far as biological differences allow, in precisely the same way as the modelled human would be under similar, non-experimental conditions. This particularly applies to the quality of anaesthesia, analgesia and care provided. Therefore, medical standards of care must be applied where appropriate and feasible, and prioritized over recommended veterinary and/or animal care standards. (Veterinary care refers to that delivered by veterinarians or under veterinary oversight. Animal care refers to regulatory conditions optimizing welfare under varying husbandry conditions.)

#### ‘Batch’ anaesthetics

Some studies may involve anaesthesia for study purposes that do affect study objectives, e.g. thoracic imaging for candidate animal selection, telemetry implantation for subsequent pharmacology studies. Such procedures are occasionally expedited by anaesthetizing animals in groups. The availability of adequately trained personnel in planning and managing anaesthesia and analgesia is pivotal to ensure safe anaesthesia on animals in ‘batch’ procedures.^17^ The practice, while convenient (and most commonly seen with small laboratory animals), is difficult to condone for three reasons: (i) group medication ignores the variability between individuals, so over- and underdosing will occur in some animals^224^; (ii) such differences will be exacerbated by differences between injection time and procedural onset; and (iii) animals anaesthetized *en masse* are unlikely to receive the level of care possible when individual animals are anaesthetized.

#### Repeated anaesthesia

Some studies require that animals be anaesthetized repeatedly, and at frequencies limited by concerns that repeated anaesthetics are somehow ‘harmful’. Such concerns are unwarranted when anaesthetic techniques are selected for safe repeatability. Prescribed inter-anaesthetic intervals are arbitrary if they fail to consider: (i) the drugs being given repeatedly; (ii) the standard of anaesthetic management provided; (iii) the procedure(s) for which the anaesthetics are required; and (iv) the duration of the repeated procedure. The intervals between which animals may be safely and humanely re-anaesthetized are minimized when short-acting drugs with non-cumulative properties are used for brief, innocuous procedures during which adverse physiological effects, e.g. hypotension and hypothermia, are prevented. Repeated anaesthetics in pigs do not appear to cause adverse effects.^225^

#### Legal considerations

Recommendations concerning anaesthesia in animal experiments must meet legal requirements. For brevity, only the EU Directive 2010/63/EU and the Animal (Scientific Procedures) Act 1986 [A(SP)A] have been considered here although other legislation, e.g., those concerned with controlled drug prescription are pertinent. Both authorities express three common themes with respect to anaesthetics and analgesics: (i) the need to control pain; (ii) the conditions of use; and (iii) competencies.

##### Pain control

The broad goals are to minimize pain, suffering and distress using general and/or local anaesthesia and/or analgesia (or, enigmatically, other ‘appropriate’ methods). Anaesthesia must be used in procedures involving serious [sic] injuries that may cause severe pain, and analgesics must be used if postprocedural pain is likely after recovery from anaesthesia. Disconcertingly, these mandates are conditional with scenarios given where anaesthesia and/or analgesia may be withheld.

##### Conditions of use

Anaesthetics need not be used when: (i) anaesthesia is judged to be more traumatic [sic] to the animal than the procedure itself; or (ii) anaesthesia, and postoperative analgesia are incompatible with procedural objectives. A strong scientific and ethical argument is required to withhold anaesthesia and/or analgesia. The Directive also requires that drugs stopping or restricting an animal’s capacity to show pain, presumably NMBAs, must not be used unless adequate levels of anaesthesia or analgesia are assured and that a scientific justification and details of the anaesthetic or analgesic regime have been provided. The A(SP)A 1986 is more meticulous in defining conditions for NMBA use (see below).

##### Staff competence

This relates to the requirements of both authorities that staff shall be adequately educated and trained before they design and/or carry out procedures on animals and/or take care of animals. The Directive of National Competent Authorities for the implementation of Directive 2010/63/EU^1^ specifies learning outcomes that are instrumental in defining both theoretical and practical competences and skills as well as the species-specific knowledge needed to perform these functions. Before carrying out these functions, staff must be supervised until they have demonstrated the requisite competence. This process will normally involve animal-free training in the theory and fundamentals of anaesthesia followed by adequate periods of supervised practical application in the defined species. In conjunction with continuing professional development, acquired skills can develop if they are applied frequently and revised, when necessary, after critical evaluation. The A(SP)A also mandates that staff possess personal licenses (PILs) which are granted on completion of formal modular training designed to meet the requirements of six procedural categories, three of which are defined in terms of the necessity for sedation, analgesia or general anaesthesia. Both A(SP)A and the Directive recognize that training and supervision are ongoing processes that require regular review, especially before implementing new knowledge such as the use of new anaesthetic or analgesic agents or methods.

However, these legal requirements have two limitations. First, they fail to allow for training in the management of unexpected, relatively uncommon but potentially fatal incidents which may occur in the course of anaesthetising sufficient numbers of ‘normal’ animals. Second, they do not accommodate anaesthetic challenges arising, for example, when new genotypes are to be anaesthetized for the first time, or when studies involving unfamiliar procedures or treatments are planned. In medical anaesthetic practice, simulation training plays a major role in resolving the first limitation, and the Royal College of Anaesthetists (RCoA) sponsor advanced initiatives in this area. Nothing equivalent exists in veterinary anaesthesia, although elements of the RCoA’s initiatives could be adapted to train uncommonly needed but life-saving skills in laboratory animal anaesthesia. The second problem, albeit uncommon, poses a conundrum: the law requires staff to be adequately educated and trained before they conduct procedures, i.e. to anaesthetize animals, but does not explain how this is accomplished when those procedures, being experimental, have never been conducted before. Similarly, one cannot be adequately trained to anaesthetize genetically modified animals if that phenotype has not previously been anaesthetized. This conundrum is solved, i.e. competency is gained, and failures minimized, by adopting an iterative process managed by highly qualified staff applying early endpoints (when needed) to ensure animal welfare. Learning through a ‘trial-and-error’ training programme with unqualified supervision is irresponsible and condemned.

In all circumstances, it is a legal imperative that measures are taken to eliminate the gap between the skills available and the skills required. The measures required depend on the size of the skill differential, the magnitude of the challenge and the availability (and cost) of qualified assistance.

#### Expert assistance

An ability to recognize personal limitations and to request assistance is a necessary competency of staff anaesthetising laboratory animals. However, under the A(SP)A, the legal responsibility for recognizing potential skill differentials lies with the project license (PPL) holder as opposed to a veterinarian or even a veterinary anaesthetist. Arguably, the AWERB or AWB have roles in anticipating difficulties with anaesthetic management before initially approving a project license application.

If potential difficulties are identified, the A(SP)A and the EU Directive require that the NVS or the DV, respectively, be consulted for advice and guidance. Depending on their own levels of expertise, these may recommend that further advice be sought and/or information gained. Several options are available.

(1) A literature review must be carried out to identify potential pitfalls and the measures required to avoid them. Suitable references may exist in the ‘background’ of project license applications. Details of anaesthetic and analgesic techniques used in animal experiments, if present, are often inadequate^226^ so direct contact with corresponding authors, and, subsequently, those directly responsible for the anaesthetic, is advised. Contact with staff involved in related studies should also be considered. Textbooks of surgical and anaesthetic techniques in farmed laboratory species are available and may provide some background information.^125-127,227,228^
(2) Requests for information may also be sent via online networks of approved, laboratory animal workers or veterinarians. Access to such networks is limited but should be achievable and conducted through the responsible NVS or DV.
(3) The advice of suitably experienced veterinary anaesthetists may be sought. Contact details for all Diplomates of the European College of Veterinary Anaesthesia and Analgesia (ECVAA) are available on the ECVAA’s website.^229^ A review of affiliations on the contact list may assist identification of suitably experienced individuals. Whilst most Diplomates will have theoretical knowledge of anaesthetic techniques in farmed animal species, and of complex surgical procedures in other species, not all will have practical experience with complex experimental procedures in pigs, small ruminants and calves. Recently, the Research Animal Anaesthesia Network (RAAN), an affiliate of the Association of Veterinary Anaesthetists, has been formed to promote and support the involvement of veterinary anaesthetists in experimental research involving animals. It aims to achieve this by fostering collaboration between experienced laboratory animal anaesthetists and scientists requiring assistance or advice in animal anaesthesia and analgesia.
(4) The advice of Diplomates of the European College of Laboratory Animal Medicine and other experienced laboratory animal personnel, e.g. named animal care and welfare officers, should be sought with respect to perioperative care, e.g. health-screening, acclimatization, nutrition, behavioural assessments and pain scoring, as these are important elements of experimental success.
(5) For assistance with unfamiliar and complex procedures which are relatively commonplace in medical practice, e.g. cardiopulmonary bypass, consultation with medical anaesthetists or technical experts, i.e. perfusionists, should be sought. However, medical specialists may anthropocentrise and fail to recognize species-specific differences, e.g. anaesthetic drug dosing requirements and pain behaviours, which may result in major problems. For this reason, medical experts should adopt an advisory role subordinate to the NVS and associated veterinary specialists.

Exactly how the available expertise is used in the first and subsequent experiments will be determined (in the UK) by the NVS and the PPL holder, but the legal requirement is for experts to attend until the nominated anaesthetist has demonstrated the requisite competence. In some experiments it may prove expeditious if the supervising expert undergo personal licensing and assume responsibility for the anaesthetic until the study’s conclusion. Some Diplomates of the ECVAA will have personal licenses and be available to accept such responsibilities at short notice. In many studies, conducting pilot studies under expert supervision on small numbers of animals to refine anaesthetic and procedural techniques beforehand should be considered. Satisfactory competencies can also be gained by increasing challenges gradually, providing the confounding effects of this process on data are recognized.

#### Controlled substances

Irrespective of the nominated anaesthetist’s background, the responsibility for regulated prescription drugs, e.g. opioids and barbiturates, in terms of storage, prescription, records of use and disposal, along with licence conditions, must be established. Responsibility will normally lie with named persons at the research establishment.

### Categorizing anaesthetic risk

Whilst licensed staff may anaesthetize animals, the involvement of specialist or qualified veterinary anaesthetists offers the opportunity for experimental refinement and staff training. To this end, and to formalise the options available when complex anaesthetics are anticipated, categorizing projects according to anaesthetic risk (Table 5) may prove useful for NVSs or DVs.

**Table 5.** Anaesthetic risk categories and recommended level of supervision.^a^

Anaesthetic Risk Category	Severity	Description and examples	Recommendation for level of supervision
1 (lowest)	Terminal or Recovery	Sedation of healthy animals, e.g. for blood sample collection or in preparation for euthanasia.	It is suggested that these do not require expert consultation. All aspects of anaesthesia can be managed by a licensed staff member. Specialists may be assigned for the purpose of training, assistance or data collection but will, except in exceptional circumstances, follow the lead of assigned staff member(s).
2 (low)	Terminal or Recovery	Anticipated short-duration anaesthesia for familiar minor surgical procedures in physiologically normal animals, e.g. embryo transfer.	
3 (moderate)	Terminal	More complex studies involving prolonged anaesthesia, invasive surgical procedures, novel technologies and extensive monitoring, e.g. toxicology studies.	Specialist consultation and/or direct study participation (until adequate competencies are achieved) is recommended.
3 (moderate)	Recovery	Either: (i) anaesthesia for physiologically normal animals undergoing surgery with the potential for increased morbidity and/or mortality, e.g. diabetic studies, vascular surgery; or (ii) anaesthesia for physiologically abnormal animals for surgical procedures, e.g. animals with an adverse phenotype.	
4 (high)	Recovery	Complex unfamiliar studies involving prolonged anaesthesia, major surgical interventions, novel technologies and extensive monitoring, e.g. cardiothoracic surgery, studies involving cardiopulmonary bypass, organ transplantation, OR procedures where considerable postoperative pain is likely.	Specialist consultation and/or direct study participation is highly recommended.

a As practiced at the Roslin Institute, University of Edinburgh. The American Society of Anesthesiologists (ASA) Physical Status Classification is not incorporated because it excludes procedural complexity and staff competence as risk factors.

### General recommendations for anaesthesia

Authoritative guidelines promoting safe anaesthesia are listed in Table 6. Four veterinary documents focus on companion animal species; their contents represent a ‘counsel of perfection’ aiming to optimize the safety of client-owned animals anaesthetized for therapeutic or diagnostic, not experimental, procedures. Therefore, some elements are irrelevant, e.g. optimizing recovery in terminal experiments, preparing for cardiopulmonary resuscitation (CPR) when cardiac arrest is an experimental endpoint. Four medical documents focus on human subjects, are stringent and detailed, but offer broad principles. Appendix H of the A(SP)A applies when NMBAs are used and is both prescriptive and detailed.^230^

**Table 6.** Published recommendations and guidelines for anaesthesia.

Authority	Title	Comments	Reference
AAGBI	AAGBI Safety Guideline; Checking Anaesthetic Equipment 2012	Medical recommendations. Stringent; covers power supply, gas supplies, suction, breathing systems, ventilators, scavenging, monitors, airway equipment.	Checking anaesthetic equipment (anaesthetists.org)^231^
AAGBI	AAGBI Safety Guideline; Immediate Post-anaesthesia Recovery 2013	Seventeen-page document directed at medical anaesthetists. Limited relevance.	Immediate post anaesthesia recovery (anaesthetists.org)^232^
AAGBI	Recommendations for Standards of Monitoring During Anaesthesia and Recovery 2021	Twelve-page set of recommendations directed at medical anaesthetists. Definitive and relevant.	Recommendations for standards of monitoring during anaesthesia and recovery 2021 (anaesthetists.org)^233^
ACVAA	Small Animal Monitoring Guidelines 2025	The 2025 guidelines include updated recommendations for monitoring circulation, oxygenation, ventilation, body temperature, neuromuscular blockade and anaesthetic depth in feline and canine patients.	https://doi.org/10.1016/j.vaa.2025.03.015 ^234^
AVA	Recommended requirements when performing general anaesthesia of dogs, cats and horses.	Lists ‘minimum’ requirements to perform safer general anaesthesia in cats, dogs and horses. Available in 7 European languages. Described as a valuable resource for veterinary practitioners across Europe.	AVA-RECOMMENDED-REQUIREMENTS-ENG.pdf^235^
AVA	Anaesthetic Safety Checklist Implementation Manual (2014)	Explanatory document for ‘Anaesthetic and Safety Checklist / Recommended Procedures’.	AVA-Checklist-Booklet-FINAL-Web-copy.pdf^236^
AVA	Anaesthetic Safety Checklist	Checklist recommended by the RCVS Practice Standards Scheme. Principle subject is companion animal species.	AVA-Anaesthetic-Safety-Checklist-FINAL-UK-WEB-copy-2-CC.pdf^236^
AVA	Guidelines for Safer Anaesthesia 2018	Authored predominantly by veterinary nurses. Principle subject is companion animals. Contains further information for veterinary staff and pet owners Three available forms: Online; explanatory booklet and checklist.	https://share.google/LGT5UogYPgHREwjmC ^237^
WHO-WFSA	World Health Organization–World Federation of Societies of Anaesthesiologists (WHO-WFSA) International Standards for a Safe Practice of Anesthesia (2018)	A 10-page document establishing medical anaesthetic practice standards with resource-limited facilities in mind. Some detailed contents on anaesthesia monitoring. Highly relevant.	WHO-WFSA International Standards for a safe Practice of Anesthesia^5^
Home Office (UK)	Guidance on the Operation of the Animals (Scientific Procedures) Act 1986. Appendix H.	Guidance on the use of NMBAs (pp. 122–124). Details legal requirements for use of NMBAs in laboratory animal species. Prescriptive, instructional, and relevant.	Guidance_on_the_Operation_of_ASPA.pdf (publishing.service.gov.uk)^238^

AAGBI, Association of Anaesthetists of Great Britain and Ireland; AVA, Association of Veterinary Anaesthetists; RCVS, Royal College of Veterinary Surgeons; WHO, World Health Organization; WFSA, World Federation of Societies of Anaesthesiologists.

Recommendations for farmed animal species anaesthetised under laboratory conditions are detailed in Tables 7–13. Table 7 lists recommendations for preoperative procedural, personnel and animal preparation, Table 8 details pre-procedural equipment preparation and Table 9 details non-provisional and provisional recommendations for perioperative emergency preparation. Table 10 lists non-provisional and provisional recommendations for sedation and general anaesthesia whereas Table 11 lists the requirements for the recovery period. Table 12 summarizes the recommendations for the humane and safe use of NMBAs.

**Table 7.** Recommendations for preoperative procedural, personnel and animal preparation.

(A) Procedures and personnel	• Review project license/approvals for procedures permissible under anaesthesia. • Review the remedial steps permissible in the event of problems arising. • Ensure team are aware of: (i) the intended procedure(s); (ii) individual responsibilities before and during the anaesthetic and during recovery. Ensure that all necessary personal licenses are in place. • Ensure Health and Safety implications are understood, e.g. volatile anaesthetics, radiation and pregnant staff. • Identify an individual responsible for anaesthesia; establish and organize the level of support for that individual. • Establish anaesthetic and analgesic plan. Consider all requirements under pharmacological and non-pharmacological factors (Table 1). Record details of proposed plan. • Ensure suitable anaesthetic record forms are available. • Identify and prepare for the most likely anaesthetic and procedural problems. Alert relevant staff to these and clarify individuals’ roles in problem resolution. • Identify staff responsible for supervising recovery. • Establish preliminary plans for recovery, with endpoints, recognizing that major changes may become necessary in the light of unexpected procedural events. • Ensure suitable recovery record forms are available, indicating items for particular attention and facilitating identification of humane endpoints.
(B) Animals	• Identify the animal(s) scheduled for anaesthesia; ensure identification is secure and unambiguous. • Named (or designated) veterinary surgeon to approve animal’s fitness for anaesthesia. • Further investigations may be prescribed if unexpected changes are found on physical examination. • Abnormalities increasing risk which cannot be stabilised or resolved should prompt animal’s exclusion from study. • Identify records of individual animals’ pre-procedural (baseline) food and water intake. • Record animal’s body mass. • Prescribe duration of preoperative food and water deprivation.

See the PREPARE Guidelines for a more extensive list of experimental prerequisites.^3^

**Table 8.** Recommendations for preoperative equipment preparation.

Power	• Ensure electrically operated equipment is plugged in and switched on. • Check charge status of battery-operated equipment.
Gas supplies	• Check main (and reserve) O_2_ supplies for integrity and adequacy. • Perform ‘tug test’ on Shrader probes in pipeline gas sources. • Check flowmeter operation. • Check emergency O_2_ bypass operates when control is activated and ceases on de-activation.
Anaesthetic machine	• Complete manufacturer’ checklist (if available). • Check O_2_ failure alarm. • Ensure other alarms (overpressure; fresh gas ingress) if present. • Ensure flowmeter control and flowmeters operational. • Ensure vaporizer is attached correctly and filled and that the filling port is closed. • Ensure adequate function of suction facility (when available).
Anaesthetic Breathing System	• Check for correct assembly and function of APL valves (if present). • In rebreathing systems, check soda-lime colour, texture and volume. • In circle systems check unidirectional valve presence and function. • Leak test (20–60 cms H_2_O) selected anaesthetic breathing system using ‘the two-bag test’. • Leak test both inner and outer limbs on coaxial systems.
Airway securing equipment	• Test cuff inflation on endotracheal tubes and laryngeal mask airways. • Check patency of all airway devices before use. • Ensure laryngoscopes are operational (batteries charged). • Ensure availability of equipment (Table 4, Column 1, anaesthetic delivery).
Monitoring Devices	• Ensure monitoring equipment is functioning, zeroed and calibrated. • Check that appropriate alarm settings are set for each device. • Ensure function of O_2_ analysers, pulse oximeters and capnographs.
Anaesthetic gas scavenging	• Ensure active anaesthetic gas scavenging system is operational and attached to the appropriate device, i.e. ventilator, anaesthetic station or breathing system.
Ventilators	• Check correct hose connections and device configuration. • Ensure adequate pressure generation during inspiratory cycle. • Check pressure relief mechanisms operate at selected pressures • Ensure inspiratory:expiratory cycling.

The listed items should be checked before the scheduled procedure date to ensure adequate time is available for: (i) the replacement and/or repair of equipment and (ii) the procuration of adequate consumable stocks. Final checks must be completed the day before the scheduled procedure date. Selected equipment must be appropriately sized for the intended subject(s).

**Table 9.** Non-provisional and provisional recommendations for perioperative emergency preparation.

Airway (Non-provisional)	Ensure availability of: • mouth gags • pharyngeal swabs • laryngoscopes (or suitable light source) • stylets or hollow bougies • lidocaine applicators • endotracheal tubes OR laryngeal masks Ensure availability of suitable (endobronchial) suction device, tubing and catheter. Equipment must be appropriately sized for the intended subject(s).
Breathing (Non-provisional)	Either: a suitable anaesthetic breathing system with O_2_ and/or medical air supply OR a self-inflating resuscitation bag OR a mechanical ventilator
Circulation (Non-provisional)	• Epinephrine^a^ • Atropine^a^ • (Additional) intravenous cannulae • Appropriate volume replacement fluids • Fluid administration set • Antagonists, e.g. atipamezole, naloxone and anticholinesterase inhibitors^b^
Special (Species) Provisional	Ruminants: ruminal trocars, stomach tubes. Pigs: dantrolene (for breeds susceptible to malignant hyperthermia).
Special (Procedural)* Provisional	DC defibrillator (functional and safety tested) Surgical suction (when severe haemorrhage possible) Example: for cardiothoracic procedures: thoracostomy tubes; pericardial drains; transvenous pacing unit Other drugs, e.g. norepinephrine, dobutamine, calcium salts; glucocorticoids; heparin; sodium bicarbonate, anti-arrhythmic drugs with appropriate dose chart^a^

Not all clinically appropriate emergency interventions will be permissible under project and personal licenses (A(SP)A 1986).

a A species-specific dose-chart for these drugs should be available.

b Drugs with the potential to antagonise the analgesic effects of previously administered agents must not be used until all alternative resuscitative measures have convincingly failed. All drug-containing syringes should be suitably labelled.

**Table 10.** Minimum requirements during sedation and general anaesthesia.

Non-provisional	• Intravenous cannulation (for the infusion of fluids and/or drugs) must be achieved before the procedure begins, ideally before anaesthesia is induced. Cannulae should only be removed when IV access is no longer required. • A patent airway must be secured by appropriate means in all animals in which unconscious or deep sedation inhibit protective airway reflexes. Equipment for this purpose must be available, appropriately sized and functioning, e.g. endotracheal tube cuffs should be checked. • Inspired gases of unconscious or deeply sedated animals should be enriched with oxygen concentrations that ensure haemoglobin saturation (as indicated by pulse oximetry readings >0.92). Sufficient oxygen should be available for the procedure until that time in recovery when the animal can maintain haemoglobin saturation whilst breathing room air. • The ability and means to impose intermittent positive pressure ventilation (IPPV), either manually or mechanically, must be available. • Analgesic drugs (or techniques) should be given (or applied) before noxious procedures are begun.
Provisional	• The nominated anaesthetist must not assume other responsibilities, e.g. surgical assistance, detracting from animal safety and must remain with deeply sedated and unconscious animals until recovery endpoints are reached. • An appropriate anaesthetic breathing system must be used, and with gas flows (and/or chemical absorbent) sufficient to prevent CO_2_ rebreathing for the duration of the procedure. • Intravenous fluids should be infused if the procedure’s duration and/or invasiveness are likely to cause hypovolaemia. • A suitable anaesthetic record^a^ should be recorded.

a See AVA anaesthetic records for low-risk^235^ and high-risk^236^ cases.

**Table 11.** Recommendations for planning and early recovery from anaesthesia.

(A) Pre-recovery	Formulate a pain scoring system for use after noxious procedures which incorporates thresholds triggering ‘rescue analgesia’ administration. Rescue analgesics must be specified in terms of drug, dose and route of administration, and actions to be taken, e.g. additional expertise sought, should pain persist. Ensure suitable recovery record forms are available (Table 7(A)). These may incorporate the pain scoring system. Establish details of other interventions, e.g. antibiotic administration and wound examination. Identify and communicate major recovery concerns to staff involved in recovery supervision. Establish when additional assistance and/or NVS (or DVS) notification is required. Optimize the early recovery environment in terms of ambient temperature, noise, lighting, ventilation, dust levels and cleanliness. Ensure bedding (type, adequacy, non-palatability, dustiness) is optimal. Take measures to prevent: (i) unnecessary human stimulation, e.g. non-essential handling and interventions, casual observers; and (ii) direct animal contact and indirect animal stimulation, e.g. vocalization.
(B) Early recovery (from discontinuation of anaesthetics to restoration of vital activity)	Modify the initial pain management (recognition, quantification and treatment) plan according to procedural events. Maintain support of ventilation, airway, oxygenation, circulatory volume, tissue perfusion and body temperature until endpoints are reached, examples include the following. • End ventilatory support if spontaneous breathing appears adequate and maintains: (i) end-tidal CO_2_ concentrations <6.6 kPa (50 mmHg) and/or; (ii) minute volume of ventilation >200 ml kg^–1^ body mass. • Extubate trachea when laryngo-spinal reflexes and/or coughing are active and persistent. • End O_2_ delivery, e.g. by face mask, when SpO_2_ remains >0.92 for 5 minutes with room air. • End fluid administration when haemodynamic variables are satisfactory and stable. • Withdraw active warming (or cooling) when core temperature is within 1–2°C of normal. Remove redundant monitoring applications, e.g.: • arterial cannulae when animal begins moving or when non-invasive blood pressure measurement suffices; • ECG pads upon movement and/or no arrythmias are present; • IV cannulae when animal drinks spontaneously (and blood samples or IV administration of drugs are not required). Maintain constant supervision until vital activity is restored, the animal can stand and ambulate (and ruminants have eructated). Establish frequency of body repositioning (if required) in recumbent animals.
(C) Late recovery (from withdrawal of physiological support to resumption of normal activity)	Establish frequency of animal observation and wound(s) (if present) inspection. Offer food and water and observe responses. Record intake of food and water over suitable intervals. Continue pain assessment and treatment until deemed unnecessary. Establish body mass once animal ambulates without pain. Observe eliminative, grooming and kinetic behaviours. Record all observations.^236^ Establish criteria for: (i) animal’s discharge from recovery; and (ii) reintroduced to pen-mates.

**Table 12.** Recommendations for NMBA use in farmed animal species undergoing non-recovery and recovery procedures.

Preparation	Permissions allowing NMBAs to be used without full and effective anaesthesia and analgesia must be critically examined, confirmed and the conditions met. (This applies to both recovery and non-recovery experiments). The permitted use of NMBAs in experiments must be confirmed by pre-procedural personal and project license inspection.
Anaesthetic technique	Full and effective anaesthesia and analgesia as determined in the project licence (or EU equivalent) must be established before NMBAs are used. When anaesthesia is maintained with volatile anaesthetics end-tidal anaesthetic concentrations should be measured continuously. End-tidal concentrations must exceed published MAC values relevant to the species, procedure and anaesthetic agent used. The infusion rate of anaesthetics used in total intravenous techniques must exceed published minimum infusion rate (MIR) values relevant to the species, procedure and anaesthetic agent used. Physiological variables indicating nociceptive responses during inadequate anaesthesia/analgesia, e.g. heart rate and arterial blood pressure, must be monitored continuously until the effects of NMBAs have demonstrably ended. The use of ‘depth of anaesthesia’ monitors, e.g. the bispectral index (BIS) is recommended, particularly when experimental conditions interfere with efferent autonomic nervous activity, e.g. the use of beta-blocking drugs. Measures must be taken to ensure the immediate restoration of anaesthesia/analgesia if these become inadequate. This will involve secure patent intravenous access, and adequate doses of anaesthetics and/or analgesics which act within one injection-site brain circulation time. Suitable anaesthetics are propofol and alfaxalone; suitable analgesics are alfentanil or fentanyl. It is strongly recommended that IPPV (manual or mechanical) is imposed whenever animals are given NMBAs. When ill-advised attempts are made to spare ventilatory capacity while paralysing specific muscle groups, e.g. the extra-ocular muscles, the continuous monitoring of ventilatory function and the means of achieving immediate tracheal intubation and IPPV must be available. Measures to effectively maintain body temperature must be taken before NMBAs are administered. Anticholinesterase and antimuscarinic drugs, or suggamadex should be available to antagonise neuromuscular blockade.
Personnel	NMBAs must only be administered by experienced licensees who are competent in animal anaesthesia and aware of the actions and interactions of the drugs to be used in the target species. Animals given NMBAs must be continuously attended by experienced licensees to ensure adequate anaesthesia, analgesia (and lung ventilation) are maintained until that time in recovery when there is minimal risk of recurarization.
Equipment	Equipment for the elective or rapid (emergency) establishment of a secure airway and lung ventilation must be immediately available (see Table 9, Airway and breathing). Adequate volumes of medical gases and anaesthetics must be available for at least the intended duration of neuromuscular blockade and lung ventilation. Contingency plans must be made in the event of power failure. The batteries of electrical equipment required to ensure unconsciousness, analgesia and lung ventilation, e.g., syringe drivers, physiological monitors and ventilators must be fully charged before use.

**Table 13.** Published recommendations and guidelines for monitoring anaesthesia.

Authority	Title/synopsis	Reference
AAGBI	AAGBI Safety Guideline; Checking Anaesthetic Equipment (2012).	Checking anaesthetic equipment (anaesthetists.org)^239^
	Detailed 15-page document focused on medical anaesthetic practice. Relevant information on monitoring equipment is found on page 15.	
AAGBI	Recommendations for Standards of Monitoring During Anaesthesia and Recovery (2021).	Recommendations for standards of monitoring during anaesthesia and recovery 2021 (anaesthetists.org)^240^
	Twelve-page set of recommendations directed at medical anaesthetists under current (UK and Ireland) medical practice standards. An excellent resource; definitive and very relevant.	
ACVAA	Recommendations for monitoring anesthetized veterinary patients (2025).	https://www.vaajournal.org/article/S1467-2987(25)00071-6/fulltext ^234^
	Five-page document focusing on monitoring companion animal anaesthesia. Relevant.	
ACVAA	Guidelines for Anaesthesia in Horses.	Guidelines for Anesthesia in Horses (acvaa.org)
	Five-page document focussing on equine anaesthesia. Cursory reference to monitoring requirements; of limited relevance.	
ANZCA	PG18(A) Guideline on monitoring during anaesthesia (2017).	PS18-Guideline-on-monitoring-during-anaesthesia (anzca.edu.au)
	Eight-page guideline document devoted to monitoring anaesthesia in humans. Succinct, authoritative and relevant.	
ASA	Standards for Basic Anaesthetic Monitoring (2020).	Standards for Basic Anesthetic Monitoring (asahq.org)
	A succinct on-line resource with unequivocal recommendations of considerable relevance.	
AVA	Recommended requirements when performing general anaesthesia of dogs, cats and horses (2008).	AVA-RECOMMENDED-REQUIREMENTS-ENG.pdf^235^
	Three-page document of some vintage. Lists ‘minimum’ requirements to perform safer general anaesthesia in cats, dogs and horses. Very limited value.	
AVA	Anaesthetic Safety Checklist Implementation Manual (2014).	AVA-Checklist-Booklet-FINAL-Web-copy.pdf^236^
	Fifteen-page ‘implementation’ document for ‘Anaesthetic and Safety Checklist/Recommended Procedures’. Focusing on companion animal anaesthesia, contains relevant advice on monitoring.	
AVA	Guidelines for Safer Anaesthesia (2018).	AVA-Safer-Anaesthesia-Guildlines-Booklet-VET-Web.pdf^241^
	Authored predominantly by veterinary nurses. Principle subject is companion animals. Of limited relevance.	
EBA	EBA recommendations for minimal monitoring during Anaesthesia and Recovery (2018).	Microsoft Word–EBA European Board of Anaesthesiology Minimal monitoring.docx (eba-uems.eu)
	A 5-page document focusing on current medical anaesthetic practice, the information contained is succinct and relevant.	
WHO-WFSA	WHO-WFSA International Standards for a Safe Practice of Anesthesia (2018).	Gelb et al.^5^
	A 10-page document establishing medical anaesthetic practice standards with resource-limited facilities in mind. Some detailed contents on anaesthesia monitoring. Highly relevant.	

AAGBI, Association of Anaesthetists of Great Britain and Ireland; ACVAA, American College of Veterinary Anesthesia and Analgesia; ANZCA, Australia and New Zealand College of Anaesthetists; ASA, American Society of Anesthesiologists; AVA, Association of Veterinary Anaesthetists; EBA, European Board of Anaesthesiology; WHO, World Health Organization; WFSA, World Federation of Societies of Anaesthesiologists.

### NMBAs

The use of NMBAs considerably complicates anaesthetic management. In preventing skeletomuscular contraction, somatic motor responses and other indices of inadequate anaesthetic ‘depth’, e.g. limb withdrawal and eye position, are lost. Furthermore, spontaneous breathing becomes ineffective or ceases. NMBAs do not affect central nervous function nor provide analgesia, so their inexpert use during noxious procedures may lead to the perception of distress and pain associated with restraint and the procedure. The usual signs of inadequate anaesthesia or inadequate analgesia, e.g. tachypnoea, vocalization and reactive movement, are lost and only meticulous monitoring of other physiological alterations, e.g. heart rate, blood pressure and lacrimation, may alert the person responsible for monitoring anaesthesia to a problem.

Directive 2010/63/EU avoids the term ‘neuromuscular blocking’ but states, ‘Member States shall ensure that animals are not given any drug to stop or restrict their showing pain without an adequate level of anaesthesia or analgesia. In these cases, a scientific justification shall be provided, accompanied by the details of the anaesthetic or analgesic regime’. This is echoed in Appendix H of the Guidance on the Operation of the A(SP)A^230^ which states that persons must not use a NMBA in the course of a regulated procedure unless the person is expressly authorized to do so by the personal licence and the project licence under which the procedure is carried out; and the agent is used in combination with such level of anaesthesia or analgesia as is determined in accordance with the project licence. The use of NMBAs without adequate anaesthesia and analgesia is permitted in extraordinary and specific cases agreed by the Secretary of State (when there is, presumably, a scientific requirement): (i) in animals rendered insentient by mechanical means, e.g. decerebration; (ii) when there is unlikely to be ‘appreciable pain or distress’; (iii) for restraint purposes for non-painful procedures in some immature forms of non-mammalian species. Table 12 lists specific recommendations based on additional stipulations from Appendix H. In describing the expert use of NMBAs in animals, it must be emphasised that while they produce variable effects in all species, there are additional unaccountable differences in sheep and pigs.^202^

### Final comments

That anaesthetics can be administered successfully, i.e. the subject ultimately recovers, by relatively untrained people may confer a sense that the process is straightforward.^242^ However, biological variation in apparently ‘normal’ animals, factors discussed previously and the dearth of information on anaesthetics in large animals (compared with traditional laboratory animal species) mean that in time, morbid or lethal outcomes are almost inevitable. When anaesthetizing animals for research purposes, there are additional requirements: a legal and ethical requirement that the animal’s welfare is optimized; and that study objectives are not obfuscated. The likelihood of meeting all objectives is probably increased by including dedicated, skilled and experienced personnel in the planning and performance of anaesthesia. Legal and ethical factors will contribute to decision making on what may be considered a suitable approach for a specific scenario.

## Part III: Monitoring anaesthesia during experimental procedures in pigs, sheep, goats and cattle

### Introduction

Anaesthesia was once the principal *refinement* of animal experiments^2^ and remains important when conducted properly. Monitoring, as a critical and long-recognized element of anaesthetic management,^243^ therefore contributes to experimental *refinement*. In producing unconsciousness, anaesthetics impair life-sustaining neuro-endocrinological reflexes. Monitoring identifies and quantifies the ensuing physiological derangements allowing the anaesthetist to limit them: at *least* to a range compatible with life, *more hopefully*, to levels minimizing postoperative complications, or *ideally*, that allow a full, complication-free recovery from anaesthesia. In limiting perioperative morbidity and mortality, appropriate anaesthesia monitoring standards are likely to contribute to a *reduction* in the numbers of experimental animals required. In studies involving data collection from anaesthetised animals, physiological monitoring is a prerequisite in limiting pharmacologic and/or physiological anaesthetic ‘noise’; minimizing variance in collected data further *reduces* the numbers of animals required to achieve scientific objectives.

Perioperative mortality data from human^244-250^ companion animal^251^ and equine^252^ populations reveal an increased risk associated with inadequate monitoring. Unsurprisingly, guidelines promoting safe anaesthesia emphasize the need for monitoring standards (Table 6). However, there are no perioperative mortality or morbidity data for pigs, sheep, cattle and goats undergoing anaesthesia in laboratories or elsewhere. Therefore, the evidence base for formulating recommendations for monitoring anaesthesia in these species under laboratory conditions does not exist. Consequently, the recommendations presented here are based: (i) on previously published guidelines referring to other species (Table 1) after; (ii) experience-based adjustment by the WG making three assumptions. The assumptions are as follows.

(1) While some anaesthetic complications are common to humans, horses, companion animals and large laboratory animals, others are species-specific.
(2 Medical and (to a lesser extent) veterinary guidelines promoting good anaesthetic practice are generally directed towards practitioners:
  (i) with defined, considerable and certified levels of expertise; while competency is a regulatory EU requirement for those anaesthetizing laboratory animals it is assumed here that there is variation in the level of this competency;
  (ii) working in facilities provisioned with a prescribed (and adequate) range of functioning anaesthetic monitoring equipment; it is assumed here that the range of equipment available in large animal laboratories is variable.

See the introductory statement describing the challenges in making applicable recommendations in the face of wide variation in practitioner competency and facility provision, etc., their resolution, and the definitions of ‘highly recommended’, ‘recommended’ and ‘suggested’.

### Recommendations

#### Responsible personnel: the ‘assigned anaesthetist’

● *Highly recommended:* A competent and licensed individual must be designated as ‘the anaesthetist’ and assume responsibility for the animal throughout the anaesthetic period or the duration of sedation (see below). ‘Responsibility’ refers to the continuous (‘continuous’ means ‘prolonged without any interruption at any time’; ‘continual’ is defined as ‘repeated regularly and frequently in steady, rapid succession’) presence of ‘the anaesthetist’ for the purpose of ensuring animal insensibility and survivability through anaesthetic and analgesic drug administration, physiological monitoring, recording and support. The designated anaesthetist cannot be the individual performing the procedure.

● *Highly recommended:* Specifically trained individual(s) must be available when NMBAs are used.

● *Highly recommended:* When ‘continuous presence’ is hazardous, e.g. during radiography, facilities for remotely observing and monitoring the patient must be available.^253^

● *Recommended:* When ‘continuous presence’ is impractical, a second trained, skilled and licensed individual should be available to assume anaesthetic responsibilities for brief (<20 minute) periods. This individual should not be the person performing the procedure. Before responsibility is transferred, a plan should be drawn up and well communicated on how to react to significant disturbances in the monitored physiological variables.

#### General anaesthesia

● *Highly recommended:* The continual evaluation of depth of anaesthesia (DOA; involving ocular position, cranial nerve areflexia, general muscle tone and including nocifensive responses, e.g. interdigital skin or coronary band clamping, where appropriate), vital and complementary signs in all species. These include: airway security (see below), continual monitoring of ruminal pressure in ruminants (adult sheep, goats and cattle), periodic evaluation of muscles under compression or tension in large animals, periodic evaluation of restraining ropes on underlying structures, including skin, periodic evaluation of nerves at risk of trauma and periodic evaluation of corneal hydration. Vital signs are: heart (or pulse) rate; pulse pressure (by palpation); respiratory rate and tidal volume by observation of the thoracic wall and/or reservoir bag excursions); and the auscultation of lung sounds. Complementary vital signs include capillary refill time (CRT), mucous membrane colour and core temperature (see below).

● *Highly recommended:* A stethoscope and thermometer (or equivalent) must always be available.

● *Recommended:* The continuous use of pulse oximetry (SpO_2_), capnography, and (invasive or non-invasive) blood pressure. When appropriate, urine output and blood loss should be measured. Invasive blood pressure must be considered in cattle and large pigs.

● *Suggested:* The continuous use of electrocardiography (ECG)

These are the minimum standards for procedures banded ‘mild’. Numerous other factors dictate the *range* of physiological variables monitored, the frequency of evaluation and the required accuracy of measurement (see below).

#### Sedation with or without local anaesthetic techniques

● *Highly recommended:* The continual monitoring of the depth of sedation (including responses to surgery) vital and complementary signs in all species. See airway security below. The continual monitoring of ruminal pressure in sheep, goats and cattle.

● *Recommended*: The continuous use of pulse oximetry.

● *Suggested:* The continuous use of NIBP and ECG. Capnography should be used during ‘deep’ sedation.

● *Suggested:* NIBP should be monitored when ruminants undergo (lumbosacral) extradural anaesthesia with lidocaine (or other local anaesthetics).

#### Local anaesthetic techniques without sedation

● *Highly recommended:* The continuous assessment of surgical-site anaesthesia throughout and after procedures.

● *Recommended:* The continuous awareness of sedation arising from systemic local anaesthetic absorption.

#### Maintaining airway patency and security

● *Highly recommended:* The continual evaluation of airway patency in all species under all conditions of impaired consciousness in which the upper airway is unsecured.

● *Highly recommended:* When an endotracheal tube (ETT) is inserted, its correct positioning must be verified by clinical assessment (stethoscopy) or direct visualization of the ETT *in situ* if possible.

● *Highly recommended:* The adequacy of cuff inflation should be checked periodically by manual lung inflation and leak testing.

● *Recommended:* Partial pressure of CO_2_ in expired gas (capnography).

● *Suggested:* The continuous confirmation of correct ETT location by capnography.

#### Monitoring lung ventilation

● *Highly recommended:* the continuous evaluation of thoracic excursions, inspiratory:expiratory patterns and respiratory rate (*f*r).

● *Recommended:* The continuous measurement, i.e. quantification, of airway pressures, tidal volume (Vt), *f*r and capnographic variables, i.e. the use of capnography.

● *Suggested:* The continuous measurement of spirometric variables.

#### Measuring inspired oxygen levels

● *Recommended:* The inspired O_2_ concentration (FiO_2_) of oxygen in the anaesthetic breathing system be measured continuously by an O_2_ analyser equipped with an audible low limit alarm (which cannot be deactivated).

#### Use of NMBAs

● *Highly recommended:* The continual monitoring of vital and complementary signs. The continuous monitoring of invasive blood pressure (IBP), end-tidal inhalation agent analysers (when volatile anaesthetics are used), SpO_2_, capnography and the ECG.

● *Recommended:* Spirometry or respirometry for evaluating respiratory muscle function during recovery from neuromuscular block.

● *Suggested:* A peripheral nerve stimulator and the means of measuring evoked responses.

#### Monitoring anaesthetic delivery, support and monitoring equipment

● *Highly recommended:* During the procedure the function of all anaesthetic delivery and support devices, including anaesthetic workstations, infusion controllers, syringe drivers, mechanical ventilators and anaesthetic breathing systems should be examined at 5- to 30-minute intervals (depending on the severity of the consequences of device failure).

#### Monitoring the procedure and operators

● *Highly recommended:* The assigned anaesthetist should evaluate the ongoing procedure at appropriate intervals.

#### Duration of monitoring and clinical responsibility

● *Highly recommended:* Monitoring begins upon administration of pre-anaesthetic medication and continues until the animal makes a full recovery, undergoes scheduled euthanasia or responsibility for anaesthesia is transferred to another, suitable individual.

#### Anaesthetic records

● *Highly recommended:* A full anaesthetic record must be completed for all cases undergoing general anaesthesia unless the brevity of the procedure renders this impractical.^254^ All monitored variables should be recorded on an anaesthetic record at appropriate intervals, i.e. vital signs every 5 minutes; core temperature every 15 minutes. All significant events, i.e. drugs provided, anaesthetic complications and fluid administration rate, and their time of occurrence must be recorded.

● *Recommended:* A record should be completed for all cases receiving procedural sedation unless the brevity of the procedure or the reactivity of the animal renders this impractical. All monitored variables should be recorded on an anaesthetic record at appropriate intervals, i.e. vital signs every 5 minutes; core temperature every 15–30 minutes. All significant events and their time of occurrence must be recorded.

● *Recommended:* Automated electronic anaesthetic record systems or data loggers should be used in conjunction with manual anaesthetic records.

### Discussion

#### Responsible personnel: the assigned anaesthetist

The assignation of a single individual as ‘the anaesthetist’ to monitor anaesthesia was unanimously regarded by the WG as the single most important recommendation in the medical and veterinary recommendations reviewed. Some proposed that such assignation should be made for ‘routine’ or ‘simple’ anaesthetics. Recommendations unanimously assert that, ‘nothing can replace properly trained personnel in anaesthesia monitoring’, ‘the use of multi-parameter monitors should be considered an aid in the provision of objective data and not as a replacement for a person’ and that all personnel monitoring anaesthesia ‘must be able to understand the monitoring that is to be used and be able to interpret the outputs produced accordingly’ (see AVA, Anaesthetic Safety Checklist Implementation Manual (2014); Table 6). Put differently, the possession of a recommended list of monitoring equipment does not represent any standard of care without its application by someone skilled in their use. In monitoring any given variable, the assigned anaesthetist must be able to rapidly: (i) differentiate true deviations from normal variability; (ii) identify deteriorating conditions through trends; (iii) predict their significance; (iv) make suitable responses. Under the A(SP)A 1986, additional training and competency criteria must be met for individuals wishing to use NMBAs.^255^

However, numerous studies have shown that adverse incidents and accidents during anaesthesia are frequently attributable to error by otherwise skilled anaesthetists.^218,256,257^ Nevertheless, monitoring reduces the risks of incidents and accidents by detecting their consequences and by giving early warning of deteriorating functions for unrelated reason.^246–250^ Monitoring also promotes the refinement of future procedures requiring anaesthesia as retrospective review of anaesthetic records provides data allowing informed decision making.

#### General anaesthesia

##### Depth of anaesthesia

Monitoring the ‘depth’ of anaesthesia through the evaluation of cranial nerve reflexes, ocular position and other signs assists in making general estimates about: (i) the animal’s likely response to surgical stimulation; and (ii) the level of physiological depression present. However, measuring important physiological signs directly, e.g. arterial blood pressure and minute volume of ventilation, are the definitive indicators of the need for support, rather than the presence and briskness, or absence of specific, potentially arbitrary reflexes. DOA monitoring in pigs, sheep, goats and cattle is described in most authoritative anaesthesia textbooks, e.g. Hall, Clarke and Trim’s *Veterinary Anaesthesia*.^125-127^

Modern multiparameter anaesthetic monitors incorporate modules measuring inspired and expired concentrations of anaesthetic vapour. End-tidal concentrations do not directly represent anaesthetic depth but are useful in comparing anaesthetic uptake with the vaporiser settings. Knowledge of the MAC of an inhaled agent is essential when interpreting this information and is especially useful: (i) when partial intravenous anaesthesia is used to decrease the requirement for inhaled agents; and (ii) when NMBAs are used.

DOA monitors transforming surface electroencephalographic (EEG) signals or auditory evoked potentials have become available, although their role has yet to be established in recommending monitoring standards for human subjects. This holds true in animals, where monitoring central nervous system depression directly and accurately is difficult. The EEG, or EEG-based derivates of cortical activity, e.g. the bispectral index (BIS), are of limited use in animals because the human-based algorithms do not accommodate, amongst other things, the variation in calvaria anatomy, skin thickness and hair coverage found in animals. That said, the value of BIS monitoring increases when neuromuscular blockade obviates the use of reflexes commonly used to assess anaesthetic depth, e.g. the palpebral reflex. The influence of anaesthetics on the EEG (and derived outputs) in pigs, sheep, goats and cattle have not been fully characterized.

##### Vital signs

Vital signs, i.e. circulation, ventilation, oxygenation and body temperature, can be continually evaluated using observation, palpation and auscultation. Continuous monitoring, which is strongly recommended, necessitates constant anaesthetist–subject contact, which may be impractical. Electronic monitors are capable of continuous monitoring and recording, but (thus far, and unlike anaesthetists) largely incapable of integrating information from the most important organ systems, i.e. the nervous, cardiovascular and respiratory systems.

##### Heart rate

The heart and/or pulse rate can be: (i) observed and palpated as an apex beat; (ii) calculated on auscultation; (iii) counted electronically from the ECG and/or pulse oximeter; or (v) from non-invasive or invasive methods of blood pressure monitoring. Of these, only the ECG gives no indication of pulsatile blood flow so does not necessarily reflect cardiac output. The heart rate should be determined continuously by palpating suitable arteries as this provides additional information, i.e. pulse rate, rhythm and quality (pulse pressure). Differences between heart and pulse rate indicate cardiac contractions with reduced stroke volume. Associated arrhythmias and/or circulating volume loss may be identified on the ECG or by recording low central venous pressures (CVPs; see below) respectively.

##### Pulse quality

The continual palpation of a peripheral pulse is highly recommended in sedated and anaesthetized animals because it assesses the pulse pressure, i.e. the difference between the systolic and diastolic pressures, which allows stroke volume estimation. The pulse pressure can also be estimated from the amplitude of pulse plethysmographs. During invasive procedures, blood pressure can be inferred by observing surgical haemorrhage and pulsations in palpable arteries.

##### Respiratory rate and volume

Ventilation must be assessed near-continuously in anaesthetized and sedated animals because respiratory arrest is a life-threatening event. Respiration can be evaluated in terms of rate, volume, rhythm and the inspiratory:expiratory effort; all are assessed by observing thoracic excursions or the reservoir bag of the anaesthetic breathing system (when observation is obscured by surgical drapes). Oesophageal stethoscopy can also be used to assess lung sounds and respiratory rate.

In all animals, but particularly ruminants, monitoring respiration (and upper airway patency) must be more meticulous when the trachea is not intubated; ruminants are at especial risk from the aspiration of saliva or regurgitated rumen contents. (Consequently, *not* intubating the trachea of animals deprived of protective upper airway reflexes is not defensible.)

Respirometers attached to the ABS measure the volume of gas inspired and expired on a breath-for-breath basis, and when measured over 60 seconds yields the minute volume. Electronic respiratory monitors operating on the temperature difference between inspired (cold) and expired (warm) gas do not indicate ventilatory adequacy. More complex respiratory monitors (capnometers, capnographs, spirometers) are discussed in the following.

##### Temperature

Hypothermia is a common complication of general anaesthesia and while core temperature rarely changes rapidly, it should be monitored continuously. Accurate thermistor probes are freely available, and rapidly positioned within the oesophagus (for measuring aortic blood temperature) or the rectum. It is recommended that core temperature be monitored during all anaesthetics, but particularly when considerable heat loss is likely, e.g. when prolonged and major surgery (laparotomy, thoracotomy) is conducted on smaller, younger animals under conditions of low ambient temperatures. If active warming devices are used to prevent hypothermia, continuous monitoring of body temperature is required to prevent unintended hyperthermia.

Monitoring core temperature is highly recommended in pigs with a familial propensity to hypermetabolic disorders, i.e. porcine stress syndrome, pale soft exudative pork and malignant hyperthermia.^258,259^

##### Complementary vital signs

Monitoring variables related to the vital signs, e.g. perfusion, allows a more complete and accurate assessment of cardiopulmonary function and is highly recommended in sedated or anaesthetized animals. Perfusion is the fundamental objective of cardiovascular activity but, being difficult to measure objectively, is estimated by the integration of information from the following methods or sources: CRT, mucous membrane colour, surgical ‘ooze’, core–periphery temperature gradients and urine output (UO).

##### Capillary refill time

This is estimated by blanching the oral mucous membrane and timing the return of colour. Normally this is less than 2 seconds. Intense vasoconstriction and/or hypotension prolongs CRT. *Ceteris paribus*, prolonged CRT indicates poor perfusion. It is recommended that the CRT be recorded every 5 minutes, the core–periphery temperature gradient every 1 hour and the UO, if measured, every 2–3 hours.

##### Mucous membrane colour

Perfused oral mucous membranes in pigs should be pink; in sheep, calves and goats they may be pigmented, light grey or brown. Perfused vulval, rectal or conjunctival mucous membranes are usually pink in all species. They become bright pink in hypercapnia (or vasodilatation due to other causes) and endotoxaemia, and blue with cyanosis (haemoglobin desaturation). In anaemic animals, cyanosis may not be evident despite desaturation. When blood pressure falls and blood vessels in the oral mucosa constrict, they become pale.

##### Capillary ooze

Examination of the surgical site may reveal capillary ‘ooze’. Bright red blood at the surgical site indicates good perfusion. Darkly coloured or slowly oozing blood reflects poor perfusion.

##### Increased core–periphery temperature gradient differences

Worsening perfusion is characterized by falls in peripheral (cutaneous) temperatures and an increased core–periphery temperature gradient difference. Cold ears and extremities may indicate that the animal is hypothermic or that warm blood is being retained in the ‘core’, i.e. being withheld from non-essential tissue. In all species, increased core–periphery temperature gradient differences are quantified by the simultaneous use of two calibrated thermistors placed at appropriate sites, e.g. over the heart base (via the oesophagus) and on the ear tip.

##### Urine output

UO measurement reflects renal perfusion and in conjunction with blood and urine analysis, quantifies renal function. It coincidentally prevents bladder overdistension during prolonged procedures which, if unrelieved, contributes to postoperative discomfort. Non-invasive bladder catheterization is facilitated in female sheep and goats using an appropriately sized colposcope. It is possible, though not straightforward, in female pigs.^260^ In male pigs, sheep, goats and cattle, the bladder can only be catheterized surgically. Catheterising the urinary bladder and measuring the collected urine volume at 1–3 hourly intervals allow UO, a surrogate measure of perfusion, to be calculated. A value in excess of 1.0 ml kg^−1^ h^−1^ is held to represent adequate renal (and other vital organ) perfusion (see below).

##### Pulse oximetry

The technology measuring oxygen (O_2_) saturation of haemoglobin in arterial blood (SaO_2_) using pulse oximetry yields SpO_2_ data. The pulse oximeter is a readily applied non-invasive measure which provides information on pulmonary function (oxygenation) and in conjunction with pulse plethysmography (and other estimates of cardiac output) O_2_ delivery to peripheral tissue. Their use involves applying the pulse oximeter probe to the animal’s tongue (sheep, goat, cattle and pig), ear (sheep and pig; not in case of lower blood pressure) or tail (pig).^261^ The vulva can also be used.

Pulse plethysmography quantifies and displays the blood volume flowing between the transmitter and sensor yielding an oscillating signal whose amplitude is proportional to the pulse pressure. In conjunction with oximetry this simultaneously monitors three important physiological variables: (i) SpO_2_; (ii) pulsatile blood through a tissue bed, i.e. mechanical cardiac activity; and (iii) the pulse rate.

Pulse oximeters are most useful: (i) in revealing an animal’s capacity to maintain Hb saturation when room-air breathing resumes at the end of anaesthesia; (ii) when ‘weaning’ animals off ventilators, when minute volumes should be as low as is consistent with SpO_2_ values >0.92; and (iii) in any procedure in which blood oxygenation may be reduced. When pulse oximeters are used, the variable pitch pulse tone and the low threshold alarm should be audible to the anaesthetist.

##### Capnography

Capnography non-invasively measures (capnometry) inspired and expired carbon dioxide (CO_2_) on a breath-by-breath basis while generating a CO_2_ concentration/time waveform (capnogram). The values generated include *f*r and expired and inspired CO_2_. The capnogram identifies partial or complete airway obstruction and the effectiveness of CO_2_ removal between breaths. The end-tidal CO_2_ value usually reflects CO_2_ levels in arterial blood and is a useful indication of adequacy of ventilation. The CO_2_ level is directly related to minute ventilation. Capnography is essential during controlled or mechanical ventilation. Capnography provides indirect information about cardiac output as it reflects pulmonary and systemic perfusion. Cardiac arrest or pulmonary thromboembolism can also be diagnosed with capnography. The use of capnography is highly recommended when anaesthetizing pigs at risk of hypermetabolic reactions.

##### Non-invasive blood pressure

Arterial blood pressure (AP) measurement provides indirect information on tissue perfusion, cardiac output and arterial elasticity. NIBP monitoring using automated oscillotonometry, i.e. the application of a suitably sized inflatable cuff to a suitable appendage is rapidly applied and used properly, offers a reasonably accurate method for intermittently monitoring systolic (SAP), mean (MAP) and diastolic (DAP) arterial blood pressure. When blood flow distal to an occlusive cuff is recorded using an ultrasonic Doppler probe, then SAPs alone are registered. Oscillotonometry can be used safely on conscious, free-roaming animals, i.e. during recovery from anaesthesia. Monitoring NIBP is highly recommended during brief procedures, when there is little justification for invasive monitoring, and whenever continuous pulse palpation is impractical. Agreement between NIBP and the gold-standard, IBP has been examined in pigs,^262,263^ sheep,^264-266^ goats^265^ and cattle^265^ and is fair when IBP is within accepted ranges.

#### Invasive blood pressure

IBP measurements provide beat-to-beat information on heart rate, systolic, mean and diastolic BP and the pulse-pressure contour (PPC) and is strongly recommended when accurate and immediate blood pressure information is required, i.e. in invasive, complex and prolonged procedures^266^ in which significant cardiovascular changes are likely, e.g. when major haemorrhage is expected. A suitable artery is cannulated and after pressure–electrical transduction the PPC is displayed. In addition to measuring the SAP and DAP (and computing MAP) analysis of the PPC allows inferences to be made about cardiac output (Qt) and systemic vascular resistance (SVR).^267^ For example, left ventricular contractility can be inferred from the steepness of the ascending limb, while stroke volume is proportional to the duration of the ejection phase. Slow, low gradient ‘diastolic runoffs’ indicate high SVR.

##### Electrocardiography

The ECG provides continuous heart rate and rhythm information but does not provide assurance that the observed electrical activity represents myocardial contractility, i.e. cardiac output. Therefore, the ECG should not be the sole monitor of cardiovascular function under any circumstances. Its use is highly recommended during arrhythmogenic procedures, e.g. cardiac catheterization.

##### Spirometry

Many modern multiparameter anaesthesia monitors incorporate spirometry modules, which simultaneously measure airway pressures, flows and volumes allowing pressure–volume plots to be displayed on a breath-by-breath basis. The interpretation of ‘spirometry loops’ enables rapid detection of changes in lung and breathing system mechanics and verifies ventilator performance.^240^

##### Central venous pressure

Passing suitable catheters via the jugular vein (ruminants) or cranial vena cava (pig) to the cranial vena caval–right atrial junction before connection to a water manometer (zeroed to the level of the manubrium sternae) allows CVP to be measured continuously. In briefest terms the CVP represents the balance between inflow (venous return or right heart preload) and outflow (right ventricular output or left atrial preload) in subjects with normal cardiac function.^268^ It is lowered in hypovolaemia, and raised in cardiac failure, pulmonary embolism, fluid over-transfusion, PPV and cardiac tamponade. It is useful when aggressive volume restoration is likely under conditions of cardiac dysfunction. Values for CVP vary considerably between individuals but within the same animal, CVP is useful for monitoring circulating volume trends during prolonged anaesthesia.

##### Blood-gas analysis

Intermittent arterial blood sampling for blood-gas analysis can be used to ‘calibrate’ capnography and pulse oximetry. Modern analysers are ‘point-of-care’ devices, i.e. small, hand-held devices using specific cassettes to meet different clinical requirements. In addition to blood gases, those used during anaesthesia also analyse sodium, potassium, chloride, bicarbonate, blood urea nitrogen, creatinine and lactate levels. Such analyses are useful in the management of prolonged anaesthetics or whenever pulmonary dysfunction is possible.

##### Cardiac output

Cardiac output can be measured in several ways, each having specific advantages and disadvantages, equipment and instrumentation requirements. In general, invasive techniques are more accurate than non-invasive techniques. Cardiac output measurement provides valuable information including heart rate, stroke volume, peripheral vascular resistance and circulating blood volume. These variables can be derived simply and non-invasively by pressure-recording analytic methods (PRAMs), i.e. high-frequency sampling and PPC analysis. PRAM methods examined in pigs showed promising results.^269^

##### Rumen pressure

Ruminal tympany is a potentially lethal complication of ruminant anaesthesia. Ruminal gas accumulation, which depends on time, the volume and type of recently ingested feed, progressively ‘splints’ the diaphragm impairing ventilation. High pressures may reduce venous return to the heart, and if unrelieved, are painful in the recovering animal.

Tympany is identified by periodically observing and palpating the abdomen, specifically the left sub-lumbar concavity (fossa) which becomes convex with time. Decreasing blood pressure coincidental with increased heart rate and inspiratory pressure (during PPV) are also indicative. (In spontaneously breathing animals, the *f*r increases as Vt falls.)

‘Bloat’ should be treated if systemic effects are present and the procedure time is prolonged. Peroral placement of a gastric tube or percutaneous trocarisation of the rumen can be performed during anaesthesia. The intraoperative relief of ruminal pressure requires precautionary measures to prevent interference with aseptic surgical technique (contamination of the surgical field, instruments and/or the surgical suite).

##### Oropharyngeal fluid

Ruminants often salivate profusely during anaesthesia, and there is a risk of passive regurgitation whenever ruminal pressure is high and rumen contents are fluid. Both increase the risk of fluid accumulation in the oropharynx. This may result in the pulmonary aspiration of foreign material when the trachea is unprotected and the head inappropriately positioned. Drainage of fluid by careful positioning of the animal’s head and neck is not always effective but should be attempted in all cases.

##### Limb position and muscle compression

In large animals (cattle and adult pigs) the position of, and tension in limbs, joints and muscles require periodic attention, particularly with respect to the surface upon which they lie. In prolonged procedures periodic limb repositioning may be needed to avoid ischaemic muscle damage and/or postoperative myalgia. Similar attention must be paid to nerves at risk of positional trauma.

##### Integumentary damage

The eyes should be checked periodically and lubricated with ophthalmic ointment to ensure the cornea remains moist and protected from injury. Tapes, chords or ropes maintaining animals in an operating position must be checked periodically to ensure cutaneous blood supply is not impaired and that underlying structures are not being damaged. The same applies to bandages or tapes used to secure the ETT *in situ.*

##### Degree of monitoring

It must be appreciated, despite the recommendations enunciated previously, that numerous factors dictate: (i) the range of physiological variables monitored; (ii) the frequency each variable is monitored; and (iii) the accuracy of measurement (and recording) required for each variable. The most important determinants are: (i) the procedure, its (ii) duration; and (iii) invasiveness; (iv) species; (v) age; (vi) the experimental objectives; and (vii) whether the study is terminal or requires the animal to recover. Additional, usually more advanced and invasive monitoring methods, e.g. intracranial pressure or Qt, are required when more complicated, invasive and/or prolonged *recovery* procedures are scheduled. A greater range of variables should be monitored, more frequently, when unfamiliar drugs or techniques are being used, or when unfamiliar genotypes are being anaesthetized. In specific models it may be necessary to monitor biochemical variables for animal management rather than scientific purpose, e.g. blood glucose in diabetic pigs. Before studies begin, it is highly recommended that the anaesthetist and research group meet to discuss which variables (if any) are to be monitored for scientific (rather than management) purposes.^3^ In the final analysis, the ‘level of monitoring’ will be influenced by equipment availability and the ability to use it.

Monitoring is necessary in non-recovery procedures to ensure that physiological changes caused by anaesthesia do not corrupt the scientific value of collected data or cause intraoperative complications.

It is recommended that the variables monitored are limited to those that are necessary. Measuring, recording, analysing and responding to every possible variable may divert attention from critical changes especially when manual record keeping is employed.

##### Sedation with or without local anaesthetic techniques

Perioperative morbidity–mortality studies have not been conducted on sedated pigs, sheep, goats or cattle, with or without additional local anaesthetics. In contrast, studies on procedurally sedated humans (with or without local anaesthesia) have justified establishing monitoring standards for this subpopulation.^240^ Similar guidelines are required in animals because there’s a general belief amongst many that work with animals that physiological monitoring is only required in unconscious subjects. Based on the (mistaken) belief that sedatives cause less physiological perturbation than general anaesthetics, sedated animals usually do not (or cannot) benefit from a secured airway, intravenous access and the closer attention, i.e. more extensive physiological monitoring, unconscious animals receive. While movement and/or intolerance of even non-invasive devices, e.g. pulse oximetry and the ECG, limits the ability to fully assess function in sedated animals, an underestimation of risk justifies the high recommendation that the DOA, vital and complementary signs are continually monitored in these by an assigned anaesthetist.

Closer attention to sedated ruminants is warranted given their propensity to ruminal tympany. The possibility of regurgitation and/or the accumulation of oropharyngeal saliva further justifies the need to continuously monitor airway patency when it is unprotected.

There is limited information on morbidity and mortality in pigs, sheep, goats and calves receiving local anaesthetics. However, NIBP is suggested when local anaesthetics are injected into the lumbosacral extradural space because this can cause fatal hypotension.^270^

##### Maintaining airway patency and security

When the trachea is not intubated in anaesthetized or profoundly sedated animals, a reduction or loss of airway patency will eventually become manifest as deteriorating SpO_2_ values and changes in other indices of hypoxia and/or hypercapnia. However, upper airway obstruction in depressed or unconscious animals is an emergency and justifies the high recommendation for continual monitoring of airway patency in these circumstances. This involves detecting observable and audible signs of dyspnoea, i.e. altered, usually a strenuous, inspiratory effort accompanied by upper airway ‘gurgling’ sounds. Such vigilance is particularly important in ruminants which are at greater risk of airway obstruction caused by saliva or regurgitated rumen contents.

When ETTs are used, their correct positioning must be verified by auscultating similar lung sounds over both hemi-thoraces or (less preferably): (i) by detecting gas expulsion from the ETT upon thoracic percussion; or, during manual lung inflation, (ii) by observing symmetrical thoracic wall movement while appreciating ‘normal’ elastic lung resistance during reservoir bag compression; or (iii) by observing ‘normal’ capnograms during capnography.

##### Monitoring lung ventilation

Positive pressure ventilation by manual or mechanical means aims to optimize gas exchange while minimizing risks of ventilator-induced lung injury. Mechanical ventilator designs vary, but the simultaneous control and measurement of airway pressure, Vt and *f*r are minimal requirements. Control of inspiratory:expiratory time ratios and end-expiratory pressures are highly desirable. These settings are adjusted to maintain normocapnia, i.e. Pe^/^CO_2_ values between 5.0 and 5.7 kPa (38–42 mmHg) using the lowest inflation pressures possible.

It is recommended that ventilator settings are confirmed by measuring ventilatory variables independently, e.g. using spirometry. Modules providing information on peak, plateau, mean and end-expiratory airway pressures, ideally as a displayed waveform, provides breath-by-breath information on thoracic wall and lung mechanics.

Monitoring blood pressure is recommended whenever PPV is likely to significantly reduce Qt.

##### Measuring inspired oxygen levels

Medical authorities recommend^5^ or highly recommend^240^ that inspired O_2_ concentration (FiO_2_) of oxygen in the patient breathing system be measured continuously by an O_2_ analyser with an audible low limit alarm always set for use. (The measurement of expired O_2_ concentrations (Fe^/^O_2_) while useful, is less important.) The positioning of the sampling port, which depends on the breathing system used, must be placed in such a position that the composition of the gas mixture delivered to the patient is monitored continuously.

Whilst adequate oxygenation must be assured during anaesthesia in all species, monitoring FiO_2_ during anaesthesia in pigs, sheep, goats and cattle has been recommended, not highly recommended, in these guidelines, because in veterinary anaesthesia: (i) low-flow anaesthesia is not commonly used; (ii) the inclusion of medical air or nitrous oxide in inspired gas is less prevalent; (iii) the neurological consequences of hypoxia are more severe in human than animal subjects; (iv) animals with hypoxia-induced neurological injury can be legitimately euthanatized.

##### The use of NMBAs

Using NMBAs during anaesthesia makes it possible for conscious animals to undergo invasive surgery without showing normal signs of nociception, i.e. movement and tachypnoea. This intolerable situation, which may not be uncommon in laboratory pigs^271^ can be avoided by monitoring (and ensuring adequate) anaesthetic delivery while closely evaluating autonomic nervous activity. Ensuring end-tidal concentrations of volatile anaesthetics (when used) are in excess, e.g. 1.1–1.3 times the reported (species- and agent-specific) values of MAC goes some way to ensuring insensibility is present.

Paralysed yet conscious animals usually respond to nocistimulation with signs of sympathetic nervous stimulation, e.g. mydriasis, tachycardia, hypertension, arrhythmias, sweating and lacrimation. However, this is not always the case in paralysed conscious humans in which the sudden appearance of pallor, hypotension, bradycardia and other signs of ‘vasovagal syncope’ also indicate inadequate anaesthesia. In any paralysed animal in which an obvious change in heart rate can be linked with intense surgical stimulation and/or a failure of anaesthetic delivery, and which is not associated with haemorrhage, hypotension, hypercapnia or hypoxaemia, should raise the suspicion of consciousness, especially when accompanied with parallel changes in blood pressure, salivation, lacrimation and ECG patterns associated with rising plasma catecholamine levels, e.g. ST-segment changes leading to premature ventricular depolarizations and ventricular tachycardia.^216^ The use of NMBAs clearly justifies the need for more meticulous, e.g. IBP rather than NIBP and the more frequent monitoring of signs of autonomic nervous activity. When NMBAs are used, it is highly recommended that the alarm thresholds on all relevant monitoring devices be set to detect slight, rather than major changes, and that the audible signal volumes are set to ‘high’.

Achieving surgical levels of neuromuscular blockade (NMBA) almost always paralyses the respiratory muscles and so PPV *must* be imposed (and appropriately monitored). The need to meticulously measure ventilatory adequacy arises during recovery from NMBA because such information defines criteria when full, and then partial ventilatory support is ended. Observing the range and pattern of diaphragmatic and thoracic wall movement is subjective, so while the trachea remains intubated, the use of spirometry or respirometry to measure Vt and Vm is highly recommended. Inspiratory manometry is also recommended. The combined use of respirometry with both pulse oximetry and capnography reveals whether ventilatory variables are associated with adequate lung function and provides more substantive information on the need (or otherwise) for continued ventilatory support. The usefulness of these variables to determine adequate ventilatory function is greatly increased if baseline values are established before NMBAs are given.

Medical authorities are unanimous in recommending methods of monitoring NMBA in human patients because: (i) residual blockade continues to be responsible for critical complications in medical anaesthetic practice; and (ii) there is considerable information linking the strength of evoked activity in the *ulnaris–adductor pollicis* nerve–muscle unit (NMU) and the ability to overcome upper airway resistance and ventilate adequately. This NMU (which adducts the thumb) only exists in *Homo sapiens* so similar information for pigs, sheep, goats and cattle does not exist. Thus, monitoring neuromuscular transmission in, for example, the *N.facialis–M.levator nasolabialis*, or the *N.ulnaris–M.carpi flexorum* NMUs (which are both feasible in pigs, sheep and goats) reflects muscle force in those units, but not the ability to breathe adequately. Given the considerable variation in sensitivity of different NMUs to NMBAs, it is possible to record full muscle function in a peripheral NMU, whilst the respiratory musculature remains paralysed. For this reason, the use of mechanomyography or acceleromyography cannot be used *alone* to judge recovery of ventilatory function after NMBA use.

These points are particularly important in sheep whose response to NMBAs differs markedly from those in other domesticated species.^202^

The increased complexity of monitoring DOA, ventilatory adequacy and neuromuscular transmission in paralysed animals, coupled with the dire consequences of ineptitude justifies the need for specialized training and ensuring the prescriptions of Appendix H of Guidance on the Operation of the Animals (Scientific Procedures) Act 1986 are met.^255^

##### Monitoring anaesthetic delivery, support and monitoring equipment

The assigned anaesthetist must check all equipment before use and set all appropriate audible alarm settings. Monitoring equipment should always be used in conjunction with careful clinical observation by the anaesthetist, as there are circumstances in which equipment may not detect unfavourable clinical developments.

When the monitors are in use, the alarms (visual and audible) must be enabled. The audible component of the alarm system should be easily heard by the anaesthetist.

The continued functioning of all anaesthetic delivery and support devices, including anaesthetic workstations, infusion controllers, syringe drivers, heater blankets, mechanical ventilators and ABSs should be periodically examined.

It is highly recommended that the anaesthetic workstation be checked every 15–30 minutes for: cylinder and pipeline pressures; the anaesthetic level in the vaporizer; and the vaporizer temperature (by palpation). It is highly recommended that the flow meter(s) and vaporizer settings and the emergency O_2_ valve position are checked continually (at least every 5 minutes).

It is highly recommended that when anaesthetics or anaesthesia adjunct drugs are being delivered by infusion controllers or syringe drivers, settings must be checked at least every 10 minutes and re-adjusted if necessary. The volume to be infused must be checked. (This also applies to intravenous fluid therapy.)

The functioning of the ABSs should be checked periodically. It is highly recommended that: (i) the degree of reservoir bag distension; (ii) the breathing system pressure; (iii) the position of the adjustable pressure limiting valve; and (iv) the ABS–ETT connection are checked at 5- to 10-minute intervals. It is recommended that the CO_2_ absorbent colour is checked every 30 minutes. It is suggested that the connection of the ABS to the common gas outlet is examined every hour.

##### Monitoring the procedure and operators

It is recommended that assigned anaesthetists should evaluate the ongoing procedure at appropriate intervals and if this is not possible, e.g. for reasons of sterility, then the operator must supply any information requested. Knowing the proximity of the procedure to the airway, large blood vessels and/or nerves allows the prediction of postoperative problems with airway patency, haemorrhage and pain (and function), respectively. Anticipating ‘stimulating’ parts of the procedure allows the preparation of ‘top-ups’ or the deepening of anaesthesia. A general appraisal of procedural trauma, including the degree of traction exerted should be conducted, as this is likely to influence postoperative analgesic requirements. Haemorrhage should be continuously assessed: bloody swabs should be retrieved and weighed. Observation of arterial pulsations at the surgical site provides information on cardiovascular performance. During thoracotomy procedures, the adequacy or otherwise of lung inflation and the development of atelectasis should be noted and corrected when appropriate. Similarly, during laparotomy procedures, e.g. for gut loop creation, bowel colour should be noted.

##### Duration of monitoring and clinical responsibility

It is highly recommended that the assigned anaesthetist should observe animals unobtrusively, ideally by CCTV, from the moment pre-anaesthetic medication is given. In poorly designed pens, pigs may entrap limbs or their nares, while ruminants may hypersalivate and/or regurgitate, necessitating prompt intervention.

It is highly recommended that the anaesthetist remains with the animal throughout anaesthesia and dedicates their attention to monitoring anaesthesia. When necessary, the anaesthetist may delegate responsibility to another competent individual for brief periods.

In recovery procedures, it is highly recommended that the anaesthetist maintains constant supervision until vital activity is restored. If necessary, another competent individual may be assigned this responsibility after being fully appraised of the animal’s condition. Recovery is a high-risk period accounting for 50–60% of anaesthetic-related deaths in companion animals^251^ and 66–100% in horses.^272^

The physiological and behavioural criteria for the restoration of vital activity are detailed described (Part II: General principles); put simply, when the animal can stand, breathe normally, ambulate, prehend food and drink.

It is highly recommended that ruminants are known to have eructated and ideally, begun ruminating and judged capable of maintaining sternal recumbency, preferably the standing position, before close surveillance is ended.

Additional staff should be available in proximity to the recovery area to assist in the event of emergencies. This is particularly important when adult cattle and large pigs are involved: physical assistance may be required, for example, to reposition entrapped or ‘cast’ animals.

#### Anaesthetic records

Maintaining an accurate anaesthetic record has numerous advantages: it facilitates the identification of trends, which may lead to crisis if uncorrected; it allows informed analysis of events so forms the basis of future technical improvement (experimental refinement); it provides a database for reviewing factors contributing to critical events, should they occur; it may also elucidate unexpected scientific outcomes, e.g. explain outliers.

The A(SP)A 1986 requires that any procedural (including anaesthetic-related) morbidity and mortality is recorded to determine the need for training [Standard Condition 20]. Under the Act, an anaesthetic record which identifies supervising and supervised anaesthetists constitutes a (required) record of the regulated procedures conducted.^255^

While manual record keeping is more time-consuming than automated electronic options, it promotes focus on the examined variable, the anaesthetised subject in general, and any monitoring device used.

Manual anaesthetic records, when used, should be formatted to allow DOA and vital signs to be recorded at least every 5 minutes, along with outputs from all monitoring devices. Records should be clear and legible with all information provided; they should clearly document a case from start to finish. Changes in responsibility for the anaesthetic must be clearly identified.

It is recommended that automated electronic anaesthetic record systems or data loggers should be used in conjunction with manual anaesthetic records. This may be prescribed for studies conducted under GLP. When contemporaneous records are difficult to keep, e.g. in emergencies, absent information should be entered retrospectively using data stored in monitoring devices.

Before electronic monitors are used, they must be checked by the assigned anaesthetist to ensure they are functioning and that all appropriate parameters and alarms have been set. This includes the cycling times or frequency of recordings. The gas sampling lines should be properly attached and free from obstruction or kinks. It is critically important to ensure the oxygen analyser, pulse oximeter and capnograph are functioning correctly and that appropriate alarm limits for all monitors are set before procedures begin.

Maintaining an anaesthetic record during procedural sedation is justified on the grounds made earlier, i.e. that sedation is not without risk. However, it is recognized that when sedation is ‘light’ the number of variables capable of being monitored may be so few as to make the exercise meaningless. Similarly, maintaining a sedative record may be considered unnecessary when very brief periods of sedation are anticipated (and realized). Under these circumstances, it is highly recommended that animals remain under close observation until fully recovered.

### Final comments

A dearth of information on morbidity and mortality, critical incidents and accidents in large laboratory animal anaesthesia makes it difficult to formulate strong and defensible recommendations on monitoring anaesthesia in laboratory pigs, sheep, cattle and goats. However, the guidelines proposed here have attempted to identify standards in this area by reviewing recommendations based on studies in humans and other non-human species and to justify those selected by describing the physiological significance of the principal variables involved. It is hoped that the recommendations made here will assist in harmonizing standards in anaesthetic care for large laboratory animals in both the UK and throughout the EU’s member states.

## Part IV: Pain assessment in pigs, sheep, goats and cattle undergoing experimental procedures

### Introduction

Assessing animal pain is challenging, but a critical prerequisite for strategic quantification and effective treatment.^273,274^ Pain management in research animals is important because: (i) it is an expected feature of experimental *refinement*^1^; (ii) pain delays recovery from procedures and may exclude individuals from study, undermining the *reduction* principle^275^; and (iii) pain may introduce unpredictable ‘noise’ which will diminish the value of scientific outcomes and compromise both *refinement and reduction* principles.^276^ The majority of the literature on pain assessment in pigs, sheep, cattle and goats focuses on neonatal to adolescent animals undergoing specific husbandry interventions while receiving limited anaesthetic or analgesic protection. Therefore, this literature is of limited value when applied to older research animals undergoing quite different procedures.^277^

See introductory statements concerning this section’s commission, aim and formulation.

Pain management involves: (i) assessment; (ii) quantification and; (iii) appropriate treatment. An inability to achieve these three components increases the risk of pain and associated suffering, and decreases the value of scientific outputs. With regard to pain treatment, a preventive, multimodal approach administered for an adequate period is required.

Pain is potentially a major confounder of scientific data. Consequently, details of pain assessment, quantification and analgesic techniques must always be reported within, or supplemental to, all publications involving noxious procedures. Details of new and/or refined techniques should be reported as standalone publications in journals likely to be read by those responsible for laboratory animal care and welfare, e.g. *Laboratory Animals* and *Journal of the American Association for Laboratory Animals*. This recommendation is in accordance with the revised (2020) ARRIVE guidelines which specifies the details to be documented: (i) the approach to animal preparation for anaesthesia and procedures; (ii) details of animal husbandry; (iii) anaesthetic and analgesic drug doses; (iv) routes and frequency of administration; (v) animal monitoring; (vi) pain assessment before, during and after the procedure; (vii) the fate of the animals at the experiment’s end; and (viii) the incidence of adverse or unexpected events.

### Pain assessment

In 2020 the International Association for the Study of Pain (IASP) revised an initial definition of pain, i.e. ‘an unpleasant sensory and emotional experience associated with, or resembling that associated with, actual or potential tissue damage’ by appending six refining statements.^278^ These addenda highlight features of pain that are most relevant to animals, i.e. that verbal description is only one of several behaviours to express pain and that an inability to communicate does not negate the possibility that pain can be experienced.^278^ Understanding the definition of pain focuses its assessment on both sensory and/or emotional components; the identification of emotional behaviours in animals is pivotal to animal pain assessment.

Pain assessment in animals must be: (i) species-specific^279^; (ii) context-specific; (iii) composite, i.e. based on a combination of qualitative and quantitative variables^280,281^; and (iv) repeated. Significant differences in the normal behaviour of pigs, sheep, cattle and goats means that understanding the normal behaviours of individuals in the research environment requires regular observation and measurement. Post-acclimatization and pre-procedural examination will facilitate interpretation of post-procedural assessments. The research environment is likely to be different to the animal’s ‘normal’ environment, especially for farmed animal species, so ensuring that baseline assessments are performed in context is also necessary. Finally, pain assessment should be performed according to a pre-established schedule that accommodates procedural severity and the time elapsing since it was completed. Twice or three-times daily assessments may be reasonable in the first 48–72 hours after a noxious procedure although more frequent inspections are required after analgesia administration to ensure treatment success. More frequent assessment is also required when responses to treatment are unsatisfactory.

In physiological and/or pharmacological studies where pain is a study requirement, pain assessment determines the appropriate pain management strategy. In other words, accurate pain assessment methods allow appropriate analgesic techniques to be developed.^282^ Pain assessment also ensures that an individual’s unique pain experience will be identified and treated accordingly. Conversely, without effective pain assessment, analgesia may be withheld because pain is overlooked or provided unnecessarily because its presence has been misidentified and/or overestimated. This is important in experiments: both pain and the side effects of analgesics can affect test results.^282^ Lastly, accurate pain assessment is required for the identification of humane endpoints and in assessing experimental severity both of which are regulatory requirements.^283^

The assessment of behavioural pain signs should be conducted sequentially, i.e. remotely (unprovoked), at the pen-side, indirectly (within the pen) and directly (by interacting with the animal).

The literature on pain assessment in pigs, sheep, cattle and goats predominantly focuses on young animals undergoing noxious ‘on farm’ husbandry procedures, such as castration or tail docking. The translatability of this information to adolescent or adult animals undergoing highly invasive experimental procedures under laboratory conditions is low. However, when the potential for translatability seems high, the quality of evidence should be considered to ensure approaches to pain assessment and management are evidence-based.

A review of the literature for pain assessment in production animals reveals methods specific for: (i) studying pain *per se*, or analgesic efficacy; and (ii) assessing incidental pain resulting from experimental procedures.

The dissemination of information from records of peri-procedural pain assessments and its mitigation (or otherwise) by analgesics and other interventions has major and widespread benefits in all spheres of laboratory animal practice. Pain records should be completed with this in mind when noxious procedures are conducted. Where information on procedure- and species-specific pain signs are unavailable, constructing pain scoring records will be difficult. In these circumstances, animals undergoing novel noxious procedures should be observed continuously postoperatively and all generally accepted pain signs recorded along with other deviations from normality (which should be regarded as a pain behaviour). The response to analgesic therapy should also be recorded. In this way, a bespoke, abbreviated pain management system will develop for use at less demanding intervals.

### Pigs

Pigs are challenging to examine as most, with the exception of minipigs, vocalize energetically and readily.^56^ Differentiating stress, pain and excitement can be difficult, especially if pain is mild to moderate. Nevertheless, a composite scoring system is a robust approach to identifying pain or other factors affecting welfare.^284,285^ An example of a proposed composite scoring system for postoperative pain assessment in pigs included five categories of subjective pain indicators scored using a modified visual analogue scale (VAS): passive observation from outside the enclosure; strength and character of response to physical contact; ambulation; vocalization; and overall dynamic and interactive assessment of pain including heart rate and respiratory rate.^285^

Pain assessment methods used in pigs are presented in Table 14. For the behavioural indicators of pain an approach to categorising these variables describes five main categories: avoidance and defensive behaviours; vocalizations; behaviours directed towards painful areas; postures and behaviours aiming to reduce stimulation of painful area; and general changes in activity, e.g. feeding, drinking, social and grooming behaviours.^281^

**Table 14.** Summary of pain assessment strategies used in pigs.^282,286,287^

	Assessment approach	Variable	Method	Feasibility of real-time assessment in research environments
Behavioural	Avoidance and defensive	Escape behaviours	Observation	High
	Behaviours directed towards painful area	Pain-related behaviours, e.g. huddling, opisthotonos, trembling and leg shaking	Observation	High
	Postures and behaviours aiming to reduce stimulation of painful area	Posture and posture changes	Observation	High
		Quantitative sensory testing	Instrumentation & observation following stimulation	Moderate: requires specialized equipment and expertise
	Vocalizations	Vocalization	Observation	Low: vocalization is not a pain-specific sign
	General activity changes feeding, drinking, social and grooming behaviours	Variation in normal behaviour	Observation	High
		Body mass	Walk-on scale	High: consider anticipated weight-gain when evaluating data
		Behaviour scores	Observation	High
Physiological		Autonomic nervous system	Auscultation or telemetry	High: may be applied using minimally invasive techniques. Surgical implantation, if required, can be combined with main experimental surgery at no additional welfare cost.
		Neurotransmitter expression, e.g. Substance P	Blood sampling	Low: sample analysis requires time; sample collection is invasive
		Cortisol and ACTH	Blood, saliva or hair sampling	Low: sample analysis requires time; sample collection is invasive
		Endogenous opioids	Blood sample	Low: not specific for pain and sample collection may be difficult
		Immune function: inflammatory mediators	Blood sample	Low: not specific for pain and sample collection may be difficult
Other		Facial grimace scales	Observation or image analysis*	Moderate: only available for piglets at present. Requires further investigation
		Nociceptive threshold testing	Instrumentation, stimulation, observation	Moderate: requires further investigation

### Sheep

As a prey species, sheep generally mask signs of all but severe pain.^274,287^ Subtle indicators of pain include bruxism, lameness, flock behaviour, abnormal posture and location and decreased feed intake.^274^ Various context-specific, i.e. the research environment and procedure, pain assessment methods in sheep exist. For example, 10 variables were evaluated in sheep which were individually housed sheep in mobility-limiting stanchions after thoracotomy for ventricular assist device implantation: posture; restlessness; heart rate; respiratory rate; response to palpation of the surgery site; kicking at abdomen or stamping feet; vocalization; bruxism; mental status; and food and water intake.^274^ Each item was scored and the sum used in a decision tree driving real-time pain management decision making. Another context-specific pain scoring system for pregnant sheep after laparotomy, hysterotomy and foetal catheterization attributed a score for: demeanour; movement; feeding/appetite; respiratory rate and character; and response to surgical site palpation.^288^ These two methods are exemplars of simple systems that can be developed as needed and ‘in house’.

More complex systems have been developed in sheep which have undergone validation, i.e. a statistical process of validation and refinement for the analysis of content validation, score distribution, multiple association, intra-observer reliability, inter-observer reliability, criterion validity, construct validity, item–total correlation, internal consistency, specificity and sensitivity, and identification of a rescue analgesic point.^289^ This system, the UNESP-Botucatu sheep acute pain scale (USAPS), which has application in research environments, requires observation in real time or of video records of sheep interacting with conspecifics, locomotion, head position, posture, activity and appetite.^289^

The various methods of pain assessment in sheep are detailed in Table 15. Behavioural indicators of pain have been distributed among five main categories: avoidance and defensive behaviours; vocalizations; behaviours directed towards the painful areas; postures and behaviours aiming to reduce painful area stimulation; and general activity changes, e.g. feeding, drinking, social and grooming behaviours.^281^

**Table 15.** Summary of pain assessment strategies used in sheep.^274,287,288,290-295^

Assessment approach	Variable	Method	Feasibility of real-time assessment in research environments
Behavioural	Locomotion and activity	Observation	High
	Posture and head position	Observation	High
	Appetite & food consumption	Observation	High
	Interaction with conspecifics	Observation	High
	Demeanour or comfort	Observation	High
	Response to palpation of surgery sites	Interaction and observation	High
Physiological	Heart rate	Auscultation or telemetry	Moderate: may require instrumentation
	Respiratory rate	Auscultation or telemetry	High: can be observed without interaction
	Electroencephalography	Instrumentation during anaesthesia	Low
	Rumination frequency	Auscultation	High
Other	Facial grimace scales	Observation or image analysis	Moderate: requires further investigation
	Nociceptive threshold testing	Instrumentation, stimulation and observation	Moderate: requires further investigation

### Cattle

Like sheep, cattle are prey in their natural environment and may not express ostentatious pain behaviours to limit vulnerability.^287^ Nevertheless, pain assessment is important for previously stated reasons. For cattle a unidimensional composite pain scale has been developed that is valid, reliable and responsive, with excellent internal consistency and discriminatory ability.^296^ The UNESP-Botucatu pain scale was developed to assess acute postoperative pain in cattle and can be contextualized to any environment.^297^ The observer scores locomotion, interactive behaviour, activity, appetite and miscellaneous behaviours including tail wagging, licking the surgical site, posture, kicking or foot stamping, pelvic limb extension, head position and lying down posture.^296^ A score is attributed to these variables and an analgesic intervention cut-off point can be defined.^296^

Methods of pain assessment in cattle are summarized in Table 16. Behavioural indicators have been categorized as for sheep (Table 15).^281^

**Table 16.** Summary of pain assessment strategies used in cattle.^230,233,298–303^

Assessment approach	Variable	Method	Feasibility of real-time assessment in research environments
Behavioural	Activity including accelerometery	Observation and/or downloading data from accelerometer	High
	Interactive behaviour	Observation	High
	Posture	Observation	High
	Appetite and food consumption	Observation	High
	Head rubbing	Observation	High
	Head shaking	Observation	High
	Palpation of surgery site	Interaction and observation	High
	Visual lameness assessment	Observation	High
	Electronic pressure mat	Movement through race over mat	Moderate
Physiological	Electroencephalography	Instrumentation during anaesthesia	Low
	Neurohormonal (Substance P, cortisol, haptoglobin, interleukins, endorphins, tumour necrosis factor) quantification	Blood sampling and analysis	Low
	Heart rate	Auscultation	Moderate
	Respiratory rate	Auscultation or observation from a distance	High
	Body weight	Movement through race onto scales	High
	Rumination frequency	Auscultation	High
Other	Nociceptive threshold testing	Instrumentation, stimulation and observation	Moderate: requires further investigation
	Infrared thermography	Restraint	Moderate: requires further investigation
	Facial grimace scale	Observation or image analysis	Moderate: requires further investigation

### Goats

There is less information on behavioural responses to pain in goats compared with sheep or cattle although these appear to be distinctly caprine. For example, goats are more likely to vocalize in response to pain. In particular, struggles and vocalizations of high intensity have been associated with pain in kids.^304^ Frequent posture changes and foot stamping have been recognized as signs of pain in adult goats.^305^ Until recently, there was no validated pain scoring scale for goats. In 2023, the UNESP Botucatu Goat Acute Pain Scale was developed and validated in animals of two breeds undergoing orchiectomy.^306^ The final version takes in consideration five items (posture, locomotion, attitude, interaction and attention to affected area), being each item scored as present or absent and suggests a cut-off point for rescue analgesia of ⩾3 of 10 as supports decision-making. A summary of pain assessment methods in goats is detailed in Table 17.

**Table 17.** Summary of pain assessment strategies used in goats.^304,306-308^

Assessment approach	Variable	Method	Feasibility of real-time assessment in research environments
Behavioural	High vocalization	Video analysis/observation	High
	Comfort	Observation	High
	Movement	Observation	High
	Flock behaviour	Observation	High
	Tails movements	Video analysis/observation	High
	Bruxism	Observation	High
	Posture	Video analysis/observation	High
	Locomotion	Video analysis/observation	High
	Attitude	Video analysis/observation	High
	Interaction	Video analysis/observation	High
	Appetite and food consumption	Video analysis/observation	High
	Attention to the affected area	Video analysis/observation	High
Physiological	Cortisol in plasma	Blood sampling and analysis	Low
	Heart rate	Interaction (stethoscope)	Moderate (requires expertise)
	Scrotal infrared temperature	Interaction and measurement	Moderate (requires specific equipment)
	Rumination frequency	Auscultation	High
Other	Visual analogue scale	Observation	High
	Mechanical nociceptive thresholds	Interaction	Moderate (requires some degree of expertise)

### Recommendations

● *Highly recommended:* When pain is not the experimental focus, i.e. when it occurs as the consequence of unavoidable experimental procedures, a plan for pain assessment must be included in the project proposal and incorporate intervention points that are agreed upon by relevant stakeholders.

● *Highly recommended*: Pain management strategies must be species-specific, context-specific and composite.

● *Highly recommended*: Pain management strategies must be described in any publication arising from the experiments.

● *Highly recommended*: When pain is the focus of the study, more sophisticated, invasive and comprehensive strategies are required, but humane endpoints must still be established. A combination of objective and subjective variables, scored from both remote observation and during interaction with the animals is useful.

● *Highly recommended:* An animal’s behaviour must be assessed at least twice before any procedure that is expected to cause pain, to provide individual baseline information. Post-procedural pain assessment should be carried out after recovery from anaesthesia and regularly or continually thereafter until pain is controlled.^285^ Pain assessment during recovery from anaesthesia may be complicated by emergence phenomena, e.g. altered behaviours and responses.

● *Recommended:* Prescriptive pain mitigation schedules, e.g. at least twice daily on the first 3–4 days and then once daily until all signs of pain have resolved. These have been recommended elsewhere^274^ but may not cater for all animals in all studies.

● *Recommended:* The numerous variables affecting signs of pain in large laboratory animals means the ideal approach would be the *ad hoc* development and validation of composite scoring systems for each study as they arise. However, this is time-consuming and requires specialist expertise.^289,309^ While scales exist for some species, their translation from farm to the research setting may be inappropriate. It is recommended that composite systems are developed, trialled and used to build understanding of how to interpret the behavioural and physiological changes that can be observed in real time to enable informed decision-making and treatment of pain in large laboratory animals.

● *Recommended:* The public dissemination of information on all aspects of large laboratory animal pain management (recognition, quantification, treatment) irrespective of success, novelty or previous findings.
